# Epstein-Barr Virus Infection of Naïve B Cells In Vitro Frequently Selects Clones with Mutated Immunoglobulin Genotypes: Implications for Virus Biology

**DOI:** 10.1371/journal.ppat.1002697

**Published:** 2012-05-10

**Authors:** Emily Heath, Noelia Begue-Pastor, Sridhar Chaganti, Debbie Croom-Carter, Claire Shannon-Lowe, Dieter Kube, Regina Feederle, Henri-Jacques Delecluse, Alan B. Rickinson, Andrew I. Bell

**Affiliations:** 1 School of Cancer Sciences, College of Medicine and Dental Sciences, University of Birmingham, Edgbaston, Birmingham, United Kingdom; 2 Department of Haematology and Oncology, Georg August University Göttingen, Göttingen, Germany; 3 Department of Virus-Associated Tumours, DKFZ, Heidelberg, Germany; University of Wisconsin-Madison, United States of America

## Abstract

Epstein-Barr virus (EBV), a lymphomagenic human herpesvirus, colonises the host through polyclonal B cell-growth-transforming infections yet establishes persistence only in IgD^+^ CD27^+^ non-switched memory (NSM) and IgD^−^ CD27^+^ switched memory (SM) B cells, not in IgD^+^ CD27^−^ naïve (N) cells. How this selectivity is achieved remains poorly understood. Here we show that purified N, NSM and SM cell preparations are equally transformable *in vitro* to lymphoblastoid cells lines (LCLs) that, despite upregulating the activation-induced cytidine deaminase (AID) enzyme necessary for Ig isotype switching and Ig gene hypermutation, still retain the surface Ig phenotype of their parental cells. However, both N- and NSM-derived lines remain inducible to Ig isotype switching by surrogate T cell signals. More importantly, IgH gene analysis of N cell infections revealed two features quite distinct from parallel mitogen-activated cultures. Firstly, following 4 weeks of EBV-driven polyclonal proliferation, individual clonotypes then become increasingly dominant; secondly, in around 35% cases these clonotypes carry Ig gene mutations which both resemble AID products and, when analysed in prospectively-harvested cultures, appear to have arisen by sequence diversification *in vitro*. Thus EBV infection *per se* can drive at least some naïve B cells to acquire Ig memory genotypes; furthermore, such cells are often favoured during an LCL's evolution to monoclonality. Extrapolating to viral infections *in vivo*, these findings could help to explain how EBV-infected cells become restricted to memory B cell subsets and why EBV-driven lymphoproliferative lesions, in primary infection and/or immunocompromised settings, so frequently involve clones with memory genotypes.

## Author Summary

Epstein-Barr virus (EBV), a growth-transforming virus linked to several B cell lymphomas in man, is usually carried as an asymptomatic latent infection in B lymphocytes. Such virus carriage selectively involves memory, but not naive, B cells. How this selectivity is achieved is poorly understood since we find that naive and memory cell types are equally susceptible to infection and growth transformation to lymphoblastoid cell lines *in vitro*. Here we ask if EBV-transformation of purified naïve B cells can induce key features of memory cells, namely immunoglobulin (Ig) class switching and Ig gene mutation. We find that EBV does not induce Ig class switching (though the infected cells remain responsive to exogenous switch signals) but can induce Ig gene mutation. Thus, within 4 weeks of infecting naive B cell preparations, one can often detect cells carrying Ig mutations which appear to have arisen by somatic hypermutation *in vitro*. Furthermore, in many cases such cells become dominant during clonal evolution of the emergent EBV-transformed cell line. Overall these findings suggest a possible explanation as to why EBV is selectively found in memory B cell populations in vivo and why EBV-positive lymphoproliferative lesions/lymphomas so frequently involve clones with mutated Ig genotypes.

## Introduction

Epstein-Barr virus (EBV), an orally transmitted herpesvirus widespread in human populations, first replicates in a permissive cell type in the oropharynx and then colonises the B cell system through a growth-transforming infection that drives the clonal expansion of latently-infected cells [1]–[3]. This growth transformation can be studied *in vitro* where infection of resting B cells occurs via CD21 receptor-mediated virus entry and leads to the outgrowth of permanent lymphoblastoid cell lines (LCLs) expressing all eight EBV latent cycle proteins (six nuclear antigens EBNAs 1, 2, 3A, 3B, 3C and –LP, and two latent membrane proteins LMPs 1 and 2) [3]. Cells displaying these same markers of viral transformation are present in the tonsillar lymphoid tissues of infectious mononucleosis (IM) patients undergoing primary EBV infection [4], [5]. Already, however, there is heterogeneity within these expanding B cell clones in IM tonsils [5], [6], with some cells apparently down-regulating viral antigen expression and switching out of cell cycle, thereby establishing a latent reservoir that can evade detection by the host T cell response. A key finding was that the cells constituting this reservoir, whether in the blood of convalescent IM patients or of long-term EBV carriers, lie within the IgD^−^ CD27^+^ memory B cell subset and not in IgD^+^ CD27^−^ naïve cells [7]–[9]. Furthermore, in IM cases where infected cell numbers were sufficient to allow single cell analysis, these cells carried somatically-mutated immunoglobulin (Ig) gene sequences typical of antigen-experienced memory cells [9], as do many of the EBV-driven lymphoproliferative disease lesions that arise in immunocompromised patients where T cell control is relaxed [10]–[14].

The physiologic process of memory selection involves IgM^+^ IgD^+^ CD27^−^ naïve B cells encountering cognate antigen in lymphoid tissues and, with antigen-specific T cell help, proliferating to form germinal centres (GCs). Here Ig variable gene sequences are subject to successive rounds of somatic hypermutation (SHM) to generate intra-clonal diversity before being re-expressed, usually in isotype-switched forms [15]. Both SHM and isotype-switching are critically dependent upon activation-induced cytidine deaminase, AID [16], [17], but are nevertheless distinct reactions that can take place independently of one another [18], [19]. The small fraction of GC progeny cells with improved affinity for antigen are then specifically selected by T cell-derived survival signals, emerging as IgD^−^ CD27^+^ memory B cells; the great majority of these are also IgM^−^ and have switched isotype to IgG or IgA, (“switched memory” cells) [20] . Given this understanding of the physiology of memory cell selection, different views have emerged as to how EBV might selectively colonise the IgD^−^ CD27^+^ memory cell pool. One view is that the virus first infects naïve cells *in vivo* and, through mimicking the activation signals normally induced by cognate antigen, drives these cells to initiate a GC reaction; the virus-infected clonal descendents of that reaction thus acquire both the genotype and phenotype of memory cells via the natural process of GC transit, albeit with virus-coded LMPs 1 and 2 substituting for affinity-based survival signals [1]. A second view, based mainly on the analysis of EBV-infected B cell clones within IM tonsillar tissues, is that memory B cells are preferentially infected, or possibly have a proliferative/survival advantage during the phase of virus-driven B cell expansion, and that their progeny subsequently re-assume memory characteristics with no requirement for GC transit [2].

An added complication to this debate is the more recent finding [21] that EBV is harboured not just in the conventional IgD^−^ CD27^+^ memory pool of healthy virus carriers but also at lower levels in a second distinct memory population comprising IgD^+^ CD27^+^ “non-switched memory” cells [20], [22], that had hitherto been largely ignored in EBV studies. Although such cells do carry somatically mutated Ig genes, opinion is divided as to whether the IgD^+^ CD27^+^ subset arises ontogenetically and is entirely independent of GC activity (as their presence in certain GC-null immune-deficiency states would imply [23], [24]) or is populated at least in part by the products of abortive/incomplete GC reactions involving Ig gene mutation without isotype switching [20], [25]–[28]. EBV's ability to colonise this subset in healthy individuals can therefore be explained in different ways depending upon one's view of non-switched memory B cell origins. Interestingly however, in T cell-deficiency states where GC development is blocked and there are no conventional switched memory B cells, EBV is sequestered in the tiny population of Ig gene-mutated, non-switched memory cells that exists in such patients rather than in the numerically dominant naive population [29], [30]. The chances of incoming virus selectively targeting such a small population by direct infection seems remote, again raising the possibility that, in naive B cells, EBV infection *per se* may be able to impose a memory genotype and/or phenotype on these cells without recourse to GC signals.

Given these uncertainties, it is surprising that little attention has been given to studying naïve, non-switched-memory and switched-memory (here designated N, NSM and SM respectively) B cell subsets as targets of EBV infection *in vitro*. Specifically, can naïve cells acquire aspects of the memory cell Ig phenotype or genotype as a result of virus transformation? Here we show that EBV infection *per se* does not alter the Ig phenotype of naive cells, although such infected cells remain susceptible to Ig class switch induction by surrogate T cell signals. However, we did find evidence for Ig gene mutation among naive B cell transformants; thus, both under limiting dilution and bulk culture conditions, selection for successful LCL outgrowth from naïve B cell infections frequently involved clones with mutated Ig genotypes that appear to have arisen through virus-induced SHM *in vitro*.

## Results

### Characteristics of purified B cell subsets

**Figure 1:**
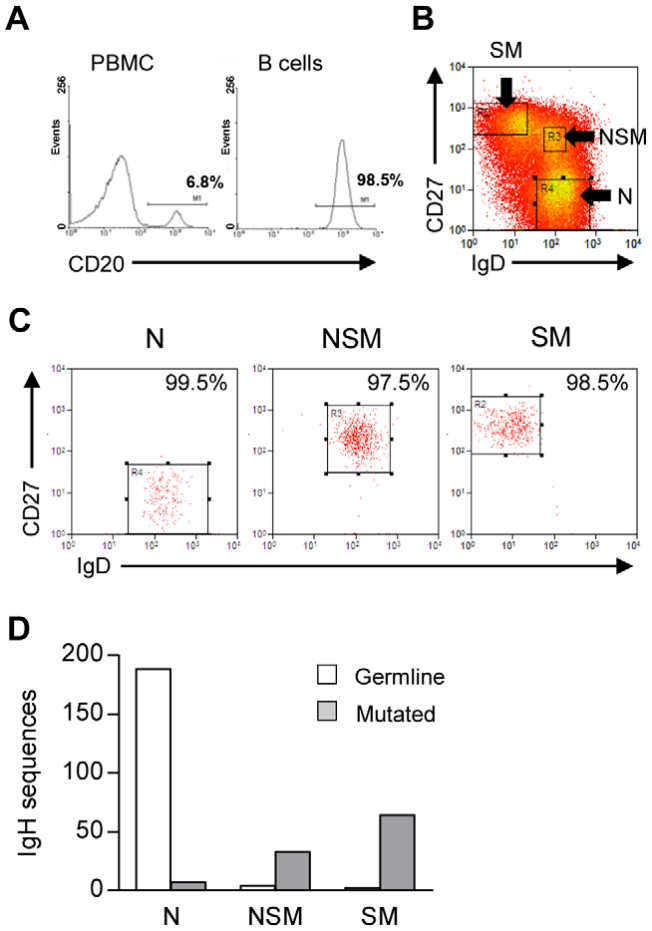
Sorting and purity of B cell subsets. (A) Isolation of CD20^+^ B cells from peripheral blood. (B) Two colour FACS analysis of B cells stained with CD27 and IgD mAbs to identify IgD^+^ CD27^−^ naïve (N), IgD^+^ CD27^+^ non-switched memory (NSM) and IgD^−^ CD27^+^ switched memory (SM) B cell populations. (C) Reanalysis to show purity of isolated N, NSM and SM B cell preparations. The figures in each panel indicate the purity of each isolated population D. Validation of cell purity by IgH analysis of sorted B cell subsets. 188 of 195 amplified IgH sequences obtained from 12 independent naive B cell preparations were classified as germline (0–2 mutations, open bars); by contrast, 33 of 37 IgH sequences from 3 independent NSM B cell preparations, and 64 of 66 IgH sequences from 6 SM B cell preparations were scored as mutated (shaded bars).

Circulating B cells, typically representing 4–8% of adult PBMC populations, were isolated by CD19 bead selection to purities consistently >98%, as shown by staining for the pan-B cell marker CD20 (Figure 1A). Such preparations were co-stained with fluorochrome-labelled Abs to IgD and CD27 in order to identify the N (IgD^+^ CD27^−^), NSM (IgD^+^CD27^+^) and SM (IgD^−^ CD27^+^) B cell subsets. Naïve B cells always represented the major subset, accounting for 60–70% of total B cell numbers, with the other two subsets each constituting between 8–25%. All three subsets were isolated to high purity by FACS sorting. Figure 1B illustrates the sort gates used and Figure 1C shows the results of re-analysing IgD and CD27 staining on the sorted populations. By these criteria, purity was always >99% for N, >96% for NSM and >98% for SM cell preparations. IgH sequence analysis further confirmed the purity of these sorts. Thus, combining results from naive cell sorts from 12 different individuals, 188 of 195 IgH sequences amplified from the N cell population were deemed to be non-mutated (i.e. less than 2 nucleotide changes from germline). In six of the above experiments, IgH sequencing was also extended to include the other subsets; as expected, the great majority of sequences amplified from the NSM and SM cell populations (33/37 and 64/66 respectively) were clearly mutated (Figure 1D).

### Infectability of purified B cell subsets

**Figure 2:**
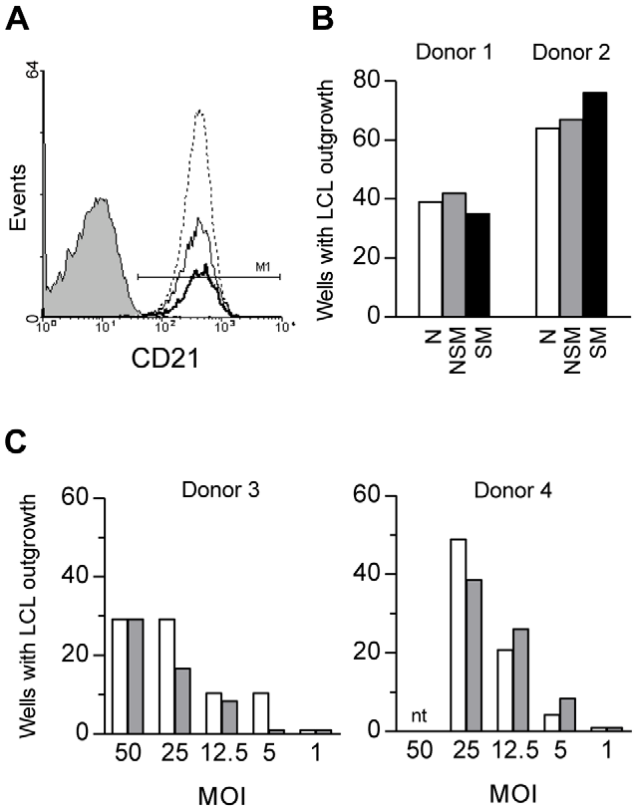
Transformation efficiency of B cell subsets. (A) FACS analysis to measure surface CD21 expression on N (dotted line), NSM (thin line) and SM (thick line) B cell subsets. Staining with isotype control reagent is shown by shading. (B) Comparison of transformation efficiencies of N, NSM and SM B cell preparations in limiting dilution cultures. Cells were exposed to EBV at 50 moi (EBV genomes per cell) for 3 h, washed and then plated at 500 cells per well into 96 replicate wells of a 96 well plate containing irradiated fibroblast feeder cells. The numbers of wells containing growth transformed foci were scored after 6 weeks. Results are shown from two representative donors. (C) Comparison of transformation efficiencies of N and NSM B cell preparations at a range of virus concentrations. Cells were exposed to EBV at the indicated moi for 3 h, washed and plated at 1000 cells per well into replicate wells of a 96 well plate. The numbers of wells showing transformation were scored after 6 weeks. Results are shown from two representative donors.

Expression of the CD21 receptor and levels of virus binding were determined for the above B cell subsets from 4 successive PBMC samples. Cell surface staining with a mAb to the EBV receptor CD21 reproducibly showed that N, NSM and SM cells express CD21 at similar levels (Figure 2A). Likewise these cells all bound virus to similar amounts, as measured by exposure to a standard virus dose at 4°C to prevent internalisation of the virus, followed by extensive washing and quantitation of EBV genome copies bound per cell by quantitative PCR (data not shown). We then assayed matched N, NSM and SM preparations for transformability using two different experimental designs. In one set of experiments, N, NSM and SM cells were exposed to a standard virus dose (50moi) before being seeded at a range of cell densities in replicate wells in 96-well plates; in other experiments, N and NSM cells were exposed to a range of virus dilutions before seeding into a 96-well plate at a standard cell number. In both cases, end points in the transformation assay were scored after 6 weeks by microscopic inspection of cultures for characteristic foci of EBV-transformed cells. Typical results from such experiments are shown in Figures 2B and 2C, and are expressed as the percentage of replicate wells scoring positive at the limiting cell seeding or limiting virus dose. Whilst absolute values for transformation efficiency varied between experiments, for any one individual donor the three B cell subsets always gave very similar yields of transformed cultures. Thereafter, cultures scoring positive on plates set up under limiting conditions were first split into duplicate wells and, where possible, further expanded over the following 3–6 weeks to yield a limiting dilution (LD)-LCL for analysis. Note that only a proportion of the wells transformed under limiting conditions could be successfully expanded in this way; overall, however, that proportion was not greatly different comparing cultures derived from N (34%), NSM (30%) and SM (24%) cell infections. We therefore conclude that the N, NSM and SM subsets from peripheral blood are equally susceptible to EBV infection and transformation *in vitro*.

### Ig phenotype of EBV-transformed B cells of N-, NSM- and SM origin

**Figure 3:**
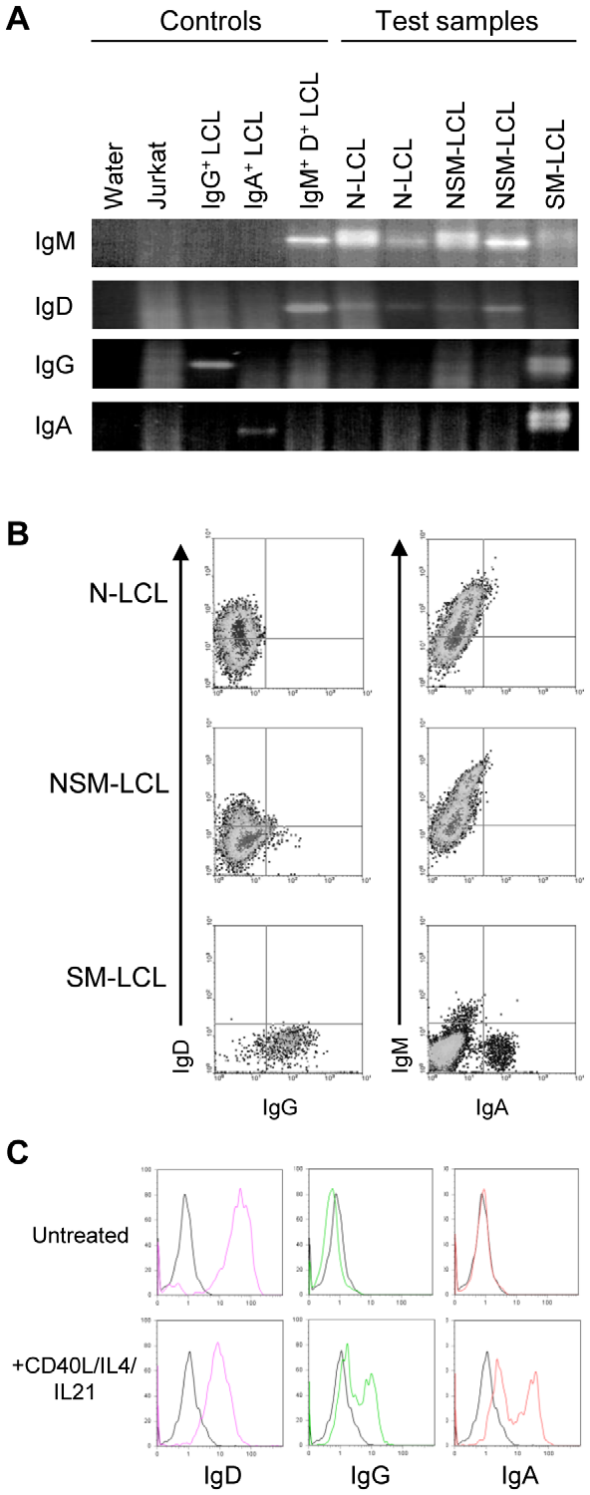
Immunophenotype of LCLs derived from different B cell subsets. (A) RT-PCR analysis of IgH gene expression. RNA from two naive (N)-LCLs, two non-switched memory (NSM)-LCLs and one switched memory (SM)-LCL was subjected to RT-PCR using a common primer in IgHV and a reverse primer specific for IgM, IgD, IgG or IgA transcripts. PCR products were analysed by agarose gel electrophoresis and visualised by ethidium bromide staining. Also shown are results from control IgG^+^, IgA^+^ and IgM^+^ IgD^+^ LCLs, while RNA from Jurkat cells was included as a negative control. (B) Two colour FACS analysis of bulk N, NSM and SM B cell-derived LCLs after dual staining cells with either RPE-labelled anti-IgD and FITC-labelled anti-IgG Abs or RPE-labelled anti-IgM and FITC-labelled anti-IgA Abs. (C) FACS staining of surface IgD, IgG and IgA in a representative N-derived LCL (initially IgD^+^ IgG^−^ IgA^−^) either untreated or exposed to CD40L/IL-4/IL21 for 7 days. Isotype control staining is shown in black lines; IgD, IgG and IgA staining shown in pink, green and red lines, respectively.

In several transformation experiments, we also set up LCLs under non-limiting conditions by infecting 2×10^6^ N, NSM and SM B cell preparations with EBV at 50moi and then culturing these cells in bulk, with subsequent expansion to bulk LCLs. To investigate whether EBV induced transformation altered Ig isotype expression in the resultant lines, we first performed RT-PCR analysis of IgH transcripts using isotype-specific primer combinations. Figure 3A shows typical results obtained, using reference B cell lines of known isotype restriction and the Ig-negative Jurkat T cell line as internal controls. Bulk lines derived from the N and NSM cell subsets were consistently positive for IgM transcripts and weakly positive for IgD but negative for the other isotypes. By contrast, bulk lines of SM cell origin expressed IgG and IgA transcripts, often accompanied by a weak signal for IgM but never for IgD; note that the weak IgM signal accords with the fact a very small proportion of cells within sorted IgD^−^ CD27^+^ memory populations are so-called “IgM-only” cells with an IgM^+^ IgD^−^ CD27^+^ phenotype [25].

The clear inference from these transcriptional data, that viral transformation had not induced detectable class switching, was strongly supported by the results of staining with mAbs specific for IgM, IgD, IgG and IgA heavy chains. Figure 3B shows typical results where both the N-derived and NSM-derived bulk LCLs retained an IgM^+^ IgD^+^ phenotype, whereas the SM-derived LCL was dominated by IgG^+^ and IgA^+^ cells. Note also that, as others have reported [31], EBV transformation induces N cells to express CD27 such that all three groups of LCLs shared a CD27^+^ phenotype (data not shown). Table 1 summarises the Ig isotype data both from bulk LCLs of N, NSM or SM origin generated as above, and from the LD-LCLs expanded from cultures of all 3 B cell subsets as described earlier. Again, all N- and NSM-derived cell lines from bulk and LD cultures were IgM^+^ IgD^+^; by contrast the SM-derived bulk lines were predominantly mixtures of IgG^+^ and IgA^+^ cells, sometimes with a small IgM+ component, whereas SM-derived LD cultures were either only IgG^+^, only IgA^+^ or mixtures of both.

**Table 1:** Ig phenotype of N-, NSM- and SM-derived LCLs.

Culture	No. tested	IgM^+^IgD^+^	IgG^+^	IgA^+^	IgM^+^IgD^−^	Mixed
N LCL, bulk	13	13	0	0	0	0
N LCL, LD† clone	82	82	0	0	0	0
NSM LCL, bulk	4	4	0	0	0	0
NSM LCL, LD† clone	8	8	0	0	0	0
SM LCL, bulk	6	0	0	0	0	6*
SM LCL, LD† clone	45	0	16	23	0	6‡

**†:** LD indicates cultures established under conditions of limiting cell numbers.

*: indicates bulk LCL cultures derived from infection of switched memory (SM) B cells and containing predominant populations of IgG^+^ and IgA^+^ cells with small numbers of IgM^+^ IgD^−^ cells.

**‡:** indicates six LD LCLs derived from infection of SM B cell and containing both IgG^+^ and IgA^+^ cells.

### Responsiveness of EBV-transformed B cells to isotype-switch signals

We then asked whether EBV-transformed cells, though not induced to undergo isotype switching by viral infection, were still capable of switching given signals that mimic physiologic T cell help. CD40L and IL-4 stimulation is known to induce isotype switching to IgG in freshly isolated N cell preparations *in vitro* [32]–[34], with switching also to IgA in the presence of IL21 [35]. Early passage bulk cultures (2–3 weeks post-infection) of N- and NSM-B cell origin were exposed to these inducing signals for 10 days; parallel cultures being maintained under normal conditions as a control. Figure 3C shows the results from an N-derived culture, typical of that seen generally with early passage cultures of N or NSM origin. Untreated cultures were uniformly IgD^+^ and lacked IgG and IgA, whereas significant fractions of cells in cultures exposed to CD40L+IL4+IL21switched to IgG or IgA, with concomitant loss of IgD.

### Ig genotype of EBV-transformed limiting dilution cultures of N, NSM and SM origin

We now examined the Ig genotype of all LD-LCLs established from the N, NSM and SM cell preparations whose pre-infection Ig genotypes had been analysed in Figure 1D. For each LD-LCL, we sequenced several cloned IgH PCR products, identified the constituent IgH V, D and J alleles and then assigned the sequences to individual CDR3 clones. By these criteria, the majority of LD-LCLs (whether derived from N, NSM or SM cells) were dominated by a single cellular clone; in addition, some were oligoclonal with 2 or 3 different clonotypes. Individual sequences within each identified clone were assigned as germline or mutated as described above.

Table 2 shows results from one such experiment, here focusing only on infections of the N cell subset. The individual clonotypes detected within each LD culture are identified through their IgH V, D and J alleles and their signature CDR3 amino acid sequence. In this experiment, of 25 cultures analysed, there were 13 monoclonal cultures (LCL6-1 to 6-13) and 2 biclonal cultures (LCL6-14, 6-15) with non-mutated genotypes. Surprisingly, however, there were also 7 monoclonal cultures with mutated genotypes (LCL6-16 to 6-22), plus 3 biclonal cultures (LCL6-23 to 6-25) with both mutated and non-mutated genotypes. Figure S1 presents the actual sequences for three of the above cultures; LCL6-2, LCL6-18 and LCL6-20 with 0, 5 and 7 mutations, respectively.

**Table 2:** IgH genotype of LD LCL clones derived by naive B cell infection (donor 6).

LD LCL	IGHV	IGHD	IGHJ	CDR3 translation	Mutations
6-1	V3-11*01	D5-24	J6*02	CAREWGGYKPLTWVDYYCGMDVW	0
6-2	V3-23*01	D6-19	J4*02	CAKDRLAVAVFWDYW	0
6-3	V4-04*02	D3-3	J3*02	CARSITIFGVVSAELDAFDIW	0
6-4	V1-08*01	D4-17	J4*02	CARGGYYEGFDYW	0
6-5	V1-69*01	D6-13	J4*02	CASASRDSSSWYERPFDYW	0
6-6	V3-48*03	D3-22	J4*02	CARDQGGVTTGAEFDYW	0
6-7	V3-23*04	D3-10	J6*03	CAKEWVGSGSYYGKPTAGYYYYMDVW	0
6-8	V4-b*01	D6-19	J4*02	CARTLVVVDW	0
6-9	V3-74*02	D3-3	J4*02	CARTRITIFGVANLDYW	0
6-10	V3-48*03	D5-5	J4*02	CARDRSRDTAMVVFDYW	0
6-11	V3-11*01	D4-17	J6*02	CARDYGDYGAWDYYYYGMDVW	0
6-12	V5-51*01	D3-10	J4*02	CARQSFGAYYFDYW	1
6-13	V4-59*01	D3-10	J4*02	CARAPAITMVRGVIEYYFDYW	2
6-14	V3-15*01	D5-12	J4*02	CTTDQWLSWEAELCW	0
	V1-69*06	D6-13	J4*02	CARDSSSSWYYFDYW	1
6-15	V4-31*01	D2-21	J6*02	CARVRLAGPLARNYYYGMDVW	1
	V5-51*01	D6-19	J5*02	CARHEEAGEVSWFDPW	1
6-16	V3-30*04	D4-23	J4*02	CAREATEVPFDSW	3
6-17	V4-4*02	D2-2	J4*02	CARGRFEEDYW	4
6-18	V1-58*01	D3-10	J5*02	CAVELWFGDIRWFDPW	5
6-19	V3-49*04	D1-26	J6*02	CTNSGSYYFYGVDVW	6
6-20	V4-59*01	D6-19	J4*02	CASGSSGWLYYFDYW	7
6-21	V3-48*03	D3-10	J4*02	CAVLGSEYSDEPFDYW	9
6-22	V1-3*01	D4-11	J6*02	CARDPRTVTRHSYYYYMDVW	9
6-23	V3-21*01	D5-12	J4*02	CARGDRGDTLRVMDYW	0
	V3-21*01	D5-12	J4*02	CARGDRGDTLRVMDYW	3
6-24	V3-21*01	D1-26	J4*02	CAREELRGYWG	0
	V1-69*01	D3-22	J4*02	CARNQDTSGSLQFDYW	3
6-25	V3-21*01	D4-17	J4*02	CARMTTVTRLVDYW	0
	V3-21*01	D3-3	J6*02	CVRAPWSGDYFYYYGLDVW	5

The above pattern of results, with a number of N-derived LD-LCLs showing significant levels of IgH mutation, was observed in 8 successive experiments, each involving a different naive B cell preparation. Combining data from all 8 experiments, we analysed 594 IgH sequences from 140 LD cultures of EBV-infected N cell preparations, and within these identified 198 distinct clones. The majority of these cultures (89/140) yielded only germline sequences, usually with a single CDR3 clonotype or in some cases with two co-resident clonotypes. However, the other 51/140 N-derived cultures yielded mutated sequences, in most cases in the absence of any detectable germline sequence. Overall, 72 of 198 (36.3%) individual clonotypes identified within these cultures were represented by mutated sequences. As a comparator, in 6 of the above experiments we also analysed 38 NSM-derived and 55 SM-derived LD cultures from infections of the memory cell subsets. Within these we identified 54 and 96 resident clones, respectively, most cultures being dominated by a single or by two co-resident clonotypes. Not surprisingly, given the predominance of mutated IgH sequences present in these B cell subsets pre-infection, all of the NSM-derived and all but two of the SM-derived cultures carried mutated IgH sequences.

**Figure 4:**
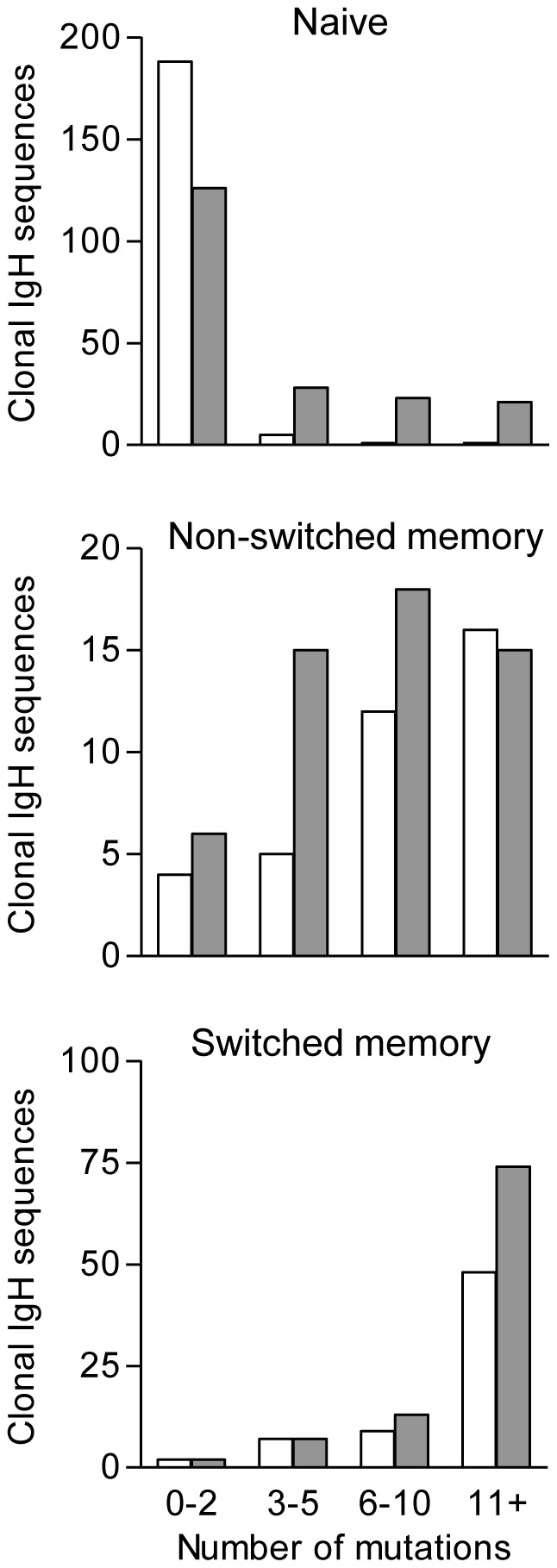
IgH mutation frequency in uninfected B cell subsets and derived LCLs. Histograms show the number of mutations among IgH clonotypes amplified from naïve, non-switched memory and switched memory B cell preparations before EBV infection (open bars) and among sequences amplified from the derived limiting dilution LCLs (shaded bars). Mutations were only scored between IgH codons 9–92 (i.e. excluding CDR3). Where LD LCL cultures yielded multiple sequences with differing numbers of mutation within the same CDR3 clone, the sequence with the maximum number of mutations was scored.

Figure 4 summarises the overall IgH genotype data from the N, NSM and SM cell preparations pre-infection (open bars) and from the derived LD-LCL cultures (shaded bars). The histograms show the total number of unique clonotypes observed in relation to their degree of divergence from germline, sequences with 0, 1 or 2 changes deemed non-mutated. Note that the degree of IgH sequence divergence among NSM-derived and SM-derived LD-LCLs is not markedly different from that within the matched pre-infection populations. More importantly, however, mutated IgH sequences are much more common in LD-LCLs derived the N cell subset than in their pre-infection counterparts.

### Characterising N-derived LD-LCLs with mutated and non-mutated Ig genotypes

**Figure 5:**
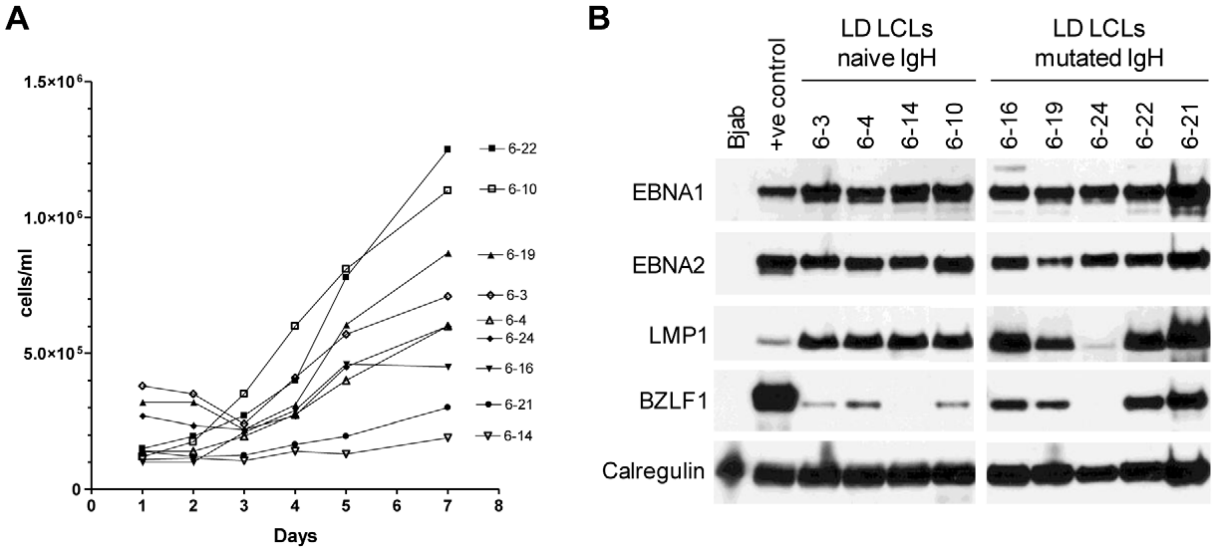
Characterisation of LCL cultures derived from naive B cells. (A). Growth rate analysis of representative LD-LCLs, all derived from the same naive B cell preparation, were measured over 7 days. LCLs with germline IgH alleles are shown by the open symbols and LCLs with mutated IgH alleles are shown by solid symbols. (B). Immunoblots showing expression of EBV antigens EBNA1, EBNA2, LMP1 and BZLF1 in the same LD-LCLs as shown in panel (A). Bjab was used as a EBV-negative control; X50/7 LCL was used as a positive control for EBNA1, EBNA2 and LMP1, while Akata-BL cells induced with anti-IgG into lytic cycle were used as the control for BZLF1 expression. Calregulin was used as a loading control.

We were interested to know whether the frequent appearance of clones with mutated Ig genotypes in LD-LCLs derived from N cell infections reflected a growth rate advantage that these cells enjoyed, perhaps one that might be linked to differences in the degree to which cells were leaving the EBV-transformed latent state and entering lytic cycle. Early passage freezings of the emergent N-derived LCLs from individual experiments were therefore taken from cryostorage and compared in proliferation assays. Figure 5A shows typical results from one such experiment comparing 5 mutated and 4 germline clones from the same donor. The emergent LD-LCLs varied considerably in their growth rates, but this was not related to their Ig genotype status; clones with mutated genotypes (closed symbols) and clones with germline genotypes (open symbols) showed a similar spread of growth rates. Furthermore, as is clear from the immunoblots in Figure 5B, neither Ig genotype status nor growth rate showed any obvious correlation with expression levels of EBV latent proteins (here illustrated using the key transformation-associated proteins EBNA1, EBNA2 and LMP1) or with the degree of lytic cycle entry as detected from levels of the immediate early lytic protein BZLF1. Thus, although cells with mutated Ig genotypes are well represented among LD-LCLs from naive cell infections, this is not because they have an inherently faster growth rate than cells carrying germline IgH sequences.

### Ig genotype of mitogen-activated versus EBV-transformed bulk cultures of N origin

The above evidence for mutated Ig genotypes in N-derived LCLs came entirely from mono- or bi-clonal populations expanded from individual limiting dilution cultures, i.e. from wells scored positive at the end of the 6 week transformation assay. Because many such wells still contained just small foci of transformed cells at that time, limitations on cell numbers precluded any prospective analysis of these cultures during their early period of expansion; as a result, sampling of LD-LCLs for genotypic analysis was often delayed until 9–12 weeks post-infection. As a second approach therefore, we turned to the analysis of resident clonotypes within bulk N cell cultures, harvesting aliquots of the same culture at regular intervals up to 12 weeks post infection. This allowed us to compare the clonal composition within an expanding EBV-infected culture with that seen in a matched culture driven to expand by repeated exposure to a non-viral proliferative trigger, CD40L/IL4. In our hands, this latter protocol induces expansions within the first 9 weeks which are at least as the equal of those driven by EBV, after which proliferation slows to a halt. Note that such mitogen-driven proliferation has never been reported to induce Ig gene mutation [36]–[38].

**Figure 6:**
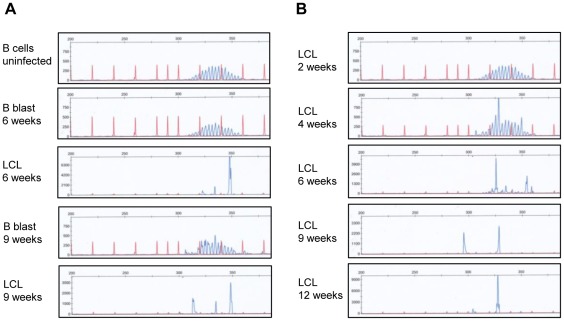
CDR3 spectratype analysis of mitogen-activated B blasts and LCL cultures derived from naive B cells. (A) Results of capillary gel electrophoresis of IgH PCR amplification products from uninfected naïve B cells, B CD40L/IL4 stimulated naive B blasts and naive B cell-derived LCLs established from donor 11 (tested at 6 and 9 weeks). A bell-shaped curve of PCR product peak sizes seen in the uninfected control culture and in the naïve B blast cultures indicates a polyclonal B cell population, while the small number of distinct peaks visible in the LCL cultures indicate that the population is dominated by a small number of clones. (B) Results of capillary gel electrophoresis of IgH PCR amplification products from naive B cell-derived LCLs established from donor 8. While a broad distribution of CDR3 lengths is observed in the uninfected control culture and 4 week LCL, only a small number of distinct peaks are visible in the LCL cultures at later time points indicating that the population is evolving towards monoclonality.

We first used the approach of IgH CDR3 spectratyping to gain an overall picture of CDR3 length distribution (i.e. clonality) in the two types of culture. Data from one such experiment are shown in Figure 6A. Clearly the CD40L/IL4-activated culture remains polyclonal despite 9 weeks of *in vitro* expansion, retaining a similar Gaussian distribution of CDR3 lengths to that seen in the original starting population. In contrast, the parallel EBV-transformed culture is dominated by sub-populations with distinct CDR3 lengths at both 6 and 9 weeks post-infection. The same samples were analysed by IgH sequencing as before, and the corresponding data are shown in Table 3. Thus all 18 sequences amplified from the initial N cell population *ex vivo* were unique and 17/18 of these were germline; likewise all 17 sequences amplified from the bulk culture after 9 weeks expansion by CD40L/IL4 were unique and non-mutated. However, the parallel EBV-transformed culture analysed at 9 weeks contained only 5 distinct clonotypes, three of which appeared from their frequent detection to represent numerically dominant cell clones. Of these three dominant clones, one (in this case, the most frequent) had a germline IgH sequence while the other two had 3–4 mutations relative to the nearest germline sequence.

**Table 3:** IgH genotype of uninfected naive B cells, mitogen stimulated naive B blasts and a bulk LCL culture derived by naive B cell infection (donor 11).

IgH sequence	IGHV	IGHD	IGHJ	CDR3 translation	Mutations
**Naive B cells**					
1	V3-48*01	D3-10	J6*02	CARARHRWFGENYYYYGMDVW	0
2	V5-a*01	D6-19	J4*02	CARPDPGAAASGWYNW	0
3	V3-21*01	D3-10	J6*02	CARDPTVWFGEFEQHHPYYYYGMDVW	0
4	V3-23*01	D2-15	J3*02	CAKDRGYCSGGSCRDAFDIW	0
5	V4-59*01	D3-9	J4*02	CARGGAGYFDWDGDYW	0
6	V5-51*01	D1-1	J6*02	CARHFTTGYYYGMDVW	0
7	V1-24*01	D3-16	J6*02	CATPWGWGIFGTYYGMDVW	0
8	V5-51*01	D6-6	J4*02	CARHASIVDYYFDYW	0
9	V5-51*01	D2-21	J4*02	CARAGRDDFLDYW	0
10	V3-23*04	D3-3	J4*02	CANLGAYDFWSGYYDLSTDW	0
11	V4-59*01	D3-22	J5*01	CARASSGYYRRFDYW	0
12	V4-39*01	D3-10	J4*02	CARHQDYYVCPPDYW	0
13	V3-48*02	D2-2	J6*02	CASKVVPAAIGYYGMDVW	0
14	V4-31*03	D5-12	J6*02	CARGLWWLQYYGMDVW	0
15	V3-07*03	D4-4	J6*02	CARGMTTVTGYYYYGMDVW	0
16	V3-07*03	D2-15	J6*02	CARDDAGMGCSGGSCYSVSYYYYSMDVW	0
17	V4-31*03	D4-17	J5*02	CASSSTVTMGWFDPW	1
18	V3-11*03	D6-19	J6*02	CARDLRVDSSGWKGDYYYGMDVW	4
**Naive B blasts**					
1	V3-15*07	D2-15	J6*02	CTTEGKPVAATQWGYYYYDMDVW	0
2	V1-46*01	D1-26	J6*02	CAGSTLVGAPWDYYYGMDVW	0
3	V4-31*03	D2-21	J5*02	CARGYCGGDCYMGGPW	0
4	V3-23*01	D4-23	J4*02	CAKEEWTTVVPGVFDYW	0
5	V5-a*01	D6-19	J4*02	CARPPKNSIAVAGARDYW	0
6	V3-21*01	D5-5	J6*02	CARDRAIDTAMWDYGMDVW	0
6	V4-31*03	D1-26	J2*01	CARDGNQWETIRW	0
7	V3-53*01	D6-19	J6*02	CARAGVGHYYYYGMDVW	0
8	V1-2*02	D1-26	J5*02	CARDLEWEPRRGNWFDPW	0
9	V3-21*01	D4-17	J4*02	CARDQGDYVVWHYFDYW	0
10	V3-21*01	D3-22	J4*02	CARDPGQPKNYYDSSGASGGYW	0
11	V6-1*01	D3-3	J4*02	CASSFGVGLDYW	0
12	V4-61*01	D4-23	J5*02	CASLSITMIVAW	1
13	V3-23*01	D4-23	J4*02	CAKVGGKRITMIVVGSW	1
14	V3-15*07	D6-6	J6*02	CTRPPSGMDVW	1
15	V4-4*07	D2-15	J6*02	CVGEVVVVAATPSYYYGMDVW	1
16	V4-31*03	D5-24	J5*02	CARDGVEMATRRAPNWFDPW	1
17	V1-69*09	D6-13	J6*02	CARPPGGGARTGYYGMDVW	1
**Bulk LCL derived from naive B cell infection**					
1	V4-31*03	D6-19	J5*02	CARVRPRIAVAGTGGWFDPW	0
2	V1-58*01	D3-16	J6*02	CAADFTFGGVIASSSYYYYGMDVW	0
3	V1-58*01	D3-16	J6*02	CAADFTFGGVIASSSYYYYGMDVW	0
4	V1-58*01	D3-16	J6*02	CAADFTFGGVIASSSYYYYGMDVW	0
5	V1-58*01	D3-16	J6*02	CAADFTFGGVIASSSYYYYGMDVW	0
6	V1-58*01	D3-16	J6*02	CAADFTFGGVIASSSYYYYGMDVW	0
7	V1-58*01	D3-16	J6*02	CAADFTFGGVIASSSYYYYGMDVW	0
8	V1-58*01	D3-16	J6*02	CAADFTFGGVIASSSYYYYGMDVW	0
9	V1-58*01	D3-16	J6*02	CAADFTFGGVIASSSYYYYGMDVW	0
9	V1-58*01	D3-16	J6*02	CAADFTFGGVIASSSYYYYGMDVW	0
10	V1-58*01	D3-16	J6*02	CAADFTFGGVIASSSYYYYGMDVW	0
11	V1-58*01	D3-16	J6*02	CAADFTFGGVIASSSYYYYGMDVW	0
12	V1-58*01	D3-16	J6*02	CAADFTFGGVIASSSYYYYGMDVW	0
13	V1-58*01	D3-16	J6*02	CAADFTFGGVIASSSYYYYGMDVW	0
14	V1-58*01	D3-16	J6*02	CAADFTFGGVIASSSYYYYGMDVW	0
15	V1-58*01	D3-16	J6*02	CAADFTFGGVIASSSYYYYGMDVW	0
16	V1-58*01	D3-16	J6*02	CAADFTFGGVIASSSYYYYGMDVW	0
17	V1-58*01	D3-16	J6*02	CAADFTFGGVIASSSYYYYGMDVW	0
18	V1-58*01	D3-16	J6*02	CAADFTFGGVIASSSYYYYGMDVW	0
19	V1-58*01	D3-16	J6*02	CAADFTFGGVIASSSYYYYGMDVW	0
20	V1-58*01	D3-16	J6*02	CAADFTFGGVIASSSYYYYGMDVW	0
21	V1-58*01	D3-16	J6*02	CAADFTFGGVIASSSYYYYGMDVW	0
22	V1-58*01	D3-16	J6*02	CAADFTFGGVIASSSYYYYGMDVW	0
23	V1-58*01	D3-16	J6*02	CAADFTFGGVIASSSYYYYGMDVW	0
24	V1-58*01	D3-16	J6*02	CAADFTFGGVIASSSYYYYGMDVW	0
25	V1-58*01	D3-16	J6*02	CAADFTFGGVIASSSYYYYGMDVW	1
26	V3-7*03	D6-13	J4*02	CALRIAAAGTRALPFDYW	2
27	V3-7*03	D6-13	J4*02	CALRIAAAGTRALPFDYW	3
28	V3-7*03	D6-13	J4*02	CALRIAAAGTRALPFDYW	3
29	V3-7*03	D6-13	J4*02	CALRIAAAGTRALPFDYW	3
30	V4-4*07	D3-22	J4*02	CARASQGYDSSGYYSYFFDYW	3
31	V4-4*07	D3-22	J4*02	CARASQGYDSSGYYSYFFDYW	3
32	V4-4*07	D3-22	J4*02	CARASQGYDSSGYYSYFFDYW	3
33	V4-4*07	D3-22	J4*02	CARASQGYDSSGYYSYFFDYW	4
34	V3-30*03	D5-5	J4*02	CAKTPRVATIMYYFDYW	5

In some experiments, greater cell yields in the N-subset sort allowed more frequent sampling of the EBV-infected bulk cultures. The CDR3 spectratyping data from one such experiment are shown in Figure 6B, clearly showing that the population remains broadly distributed in terms of CDR3 size up to 4 weeks post-infection but becomes much more focused by 6 weeks and further focused by 9 and 12 weeks. The corresponding IgH sequence data from this experiment are presented in Table 4. They show that the EBV-infected N cell culture is composed of multiple unique clones both at 2 weeks, when all amplified sequences were germline, and also at 4 weeks, at which point the first mutated sequences appear as minor components. By 6 weeks, three clonotypes (one of which was previously seen at week 4) together account for about half the amplified sequences and by 9–12 weeks the culture is dominated by just one of these clonotypes, with another remaining as a minor component. In this case, the dominant IgH clonotype in the N-derived bulk LCL is clearly mutated.

**Table 4:** Sequential analysis of the IgH genotype of a bulk LCL culture derived by naive B cell infection (donor 8).

IgH sequence	IGHV	IGHD	IGHJ	CDR3 translation	Mutations
**2 week LCL**					
1	V4-59*01	D3-10	J6*02	CARHSGITMVRPLDYYYYYGMDVW	0
2	V1-46*01	D3-16	J4*02	CARDGGFVGATDFDYW	0
3	V1-18*01	D3-9	J4*02	CARSTGGYDILTGYFPFDYW	0
4	V1-58*01	D2-2	J6*02	CAAQNDIVVVPAAMGIGYGMDVW	0
5	V4-61*01	D3-22	J5*02	CARYEYYYDSSGFYWFDPW	0
6	V4-59*01	D1-26	J1*01	CARETIVGATTAYFQHW	0
7	V1-69*01	D3-22	J2*01	CASSSGYPDWYFDLW	0
8	V5-a*01	D6-19	J4*02	CARLSGWYSESHYW	0
9	V3-15*07	D6-19	J4*02	CTTGIRIAVAGPSFDYW	0
10	V4-31*03	D5-5	J4*02	CARENNEGSPFDYW	0
11	V4-31*01	D4-17	J4*02	CARSPRLRGPYYFDYW	0
12	V1-18*01	D3-3	J6*02	CARGSYDFWSGYYDYGMDVW	0
13	V1-46*01	D3-22	J5*02	CVTDSSGFHWFDPW	0
14	V5-51*01	D5-5	J4*02	CARHVTAMADYW	0
15	V3-48*03	D2-2	J6*02	CARDGVPAAMGYYYYYGMDVW	0
16	V3-30*01	D3-10	J6*02	CARDPGVTYYYGMDVW	0
18	V4-31*03	D2-2	J4*02	CARQVAAAVDYW	0
19	V1-3*01	D1-1	J4*02	CARGPTDYW	0
20	V3-74*01	D2-15	J6*02	CARRGGSDIYYYYGMDVW	0
22	V3-48*03	D4-4	J6*02	CARDYSNYDYYYYYGMDVW	0
23	V3-23*01	D3-22	J4*02	CAKSSNYYDSSGYYSAW	0
25	V5-51*01	D3-10	J6*02	CARLYYGSGPWGMDVW	0
26	V3-30*03	D5-12	J6*02	CARAGTRYYYYGMDVW	0
29	V1-69*06	D3-10	J6*02	CASSNTVLGGDYYYGMDVW	0
21	V1-2*04	D3-3	J3*02	CARDGNLGRGDAFDIW	1
24	V4-59*01	D3-3	J4*02	CARSPPGLNNYFDYW	1
30	V5-51*01	D3-16	J6*02	CARLGAAMVFYYGMDVW	1
31	V4-39*01	D5-5	J5*02	CARPRHDITMIVSW	1
32	V1-24*01	D2-15	J6*02	CACCSRSEDYYYGMDVW	1
**4 week LCL**					
1	V4-61*01	D6-6	J4*02	CARDSSAARPDYW	0
2	V4-39*01	D2-2	J3*02	CARAGGKLVAAFDIW	0
3	V4-59*01	D3-16	J5*02	CARSGQVRFGFDPW	0
4	V1-69*01	D2-21	J6*02	CARAGGRDPPYYYYYYGMDVW	0
5	V4-59*01	D3-22	J4*02	CARAVGGPPFFNSSGYPIFDYW	0
6	V5-51*01	D6-19	J4*02	CARLQDSPPDYW	0
7	V1-46*01	D5-5	J4*02	CAREAVRYSYGHDYW	0
8	V1-f*01	D3-22	J4*02	CATARSTGYLAYW	0
9	V5-51*01	D4-4	J6*02	CARYGSYPYYYYGMDVW	0
10	V4-31*03	D5-5	J4*02	CARDGYGLDYW	1
11	V3-7*03	D1-1	J3*02	CARDTNWNGAPSAFDIW	1
12	V3-21*01	D6-13	J6*02	CARDVGSSSWYNYYGLDVW	1
13	V4-39*03	D1-26	J4*02	CARAASGSLDYW	1
14	V3-30*07	D3-22	J4*02	CAKSRYYYDSSFDYW	2
15	V3-23*04	D6-19	J4*02	CARGSSGWW	2
16	V4-59*01	D3-3	J4*02	CARVRLSGWYYFDYW	3
17	V4-4*02	D1-26	J4*02	CARDQGSYYGRWIDYW	5
18	V4-b*01	D1-7	J4*02	CARVSLEAVXFDYW	5
19	V1-46*01	D6-19	J6*02	CARDSSGWYSTGYGMDVW	6
**6 week LCL**					
1	V1-69*01	D4-23	J6*02	CARESTGDYYYYYGMDVW	0
2	V4-31*03	D5-24	J6*02	CARELKRWLQSGGGMDVW	0
3	V4-b*02	D3-22	J6*02	CARDTYYDSNGMDVW	0
4	V4-59*01	D1-26	J5*02	CARDLGSQWELGPW	0
5	V4-59*01	D1-26	J5*02	CARDLGSQWELGPW	4
6	V5-51*01	D4-23	J6*02	CARHGGGNSRYYGMDVW	0
7	V4-39*01	D4-23	J3*02	CLGGNDAFDIW	1
8	V5-51*01	D3-22	J4*02	CARQTDDSSGYYDYW	1
9	V4-59*01	D6-19	J6*02	CASMPSIAVAGDYYYYGMDVW	1
10	V3-64*05	D6-19	J6*02	CVKEGSSYYYYYYGMDVW	2
11	V3-23*04	D6-19	J4*02	CARGSSGWW	2
12	V3-23*04	D6-19	J4*02	CARGSSGWW	5
13	V3-48*02	D3-10	J6*02	CARVRGWTTYGMDVW	2
14	V3-48*02	D3-10	J6*02	CARVRGWTTYGMDVW	5
15	V3-48*02	D3-10	J6*02	CARVRGWTTYGMDVW	6
16	V3-48*02	D3-10	J6*02	CARVRGWTTYGMDVW	6
17	V3-23*04	D3-10	J6*02	CAKDRGFGELHGMDVW	2
18	V3-23*04	D3-10	J6*02	CAKDRGFGELHGMDVW	7
19	V3-23*04	D3-10	J6*02	CAKDRGFGELHGMDVW	7
20	V3-23*04	D3-10	J6*02	CAKDRGFGELHGMDVW	7
21	V3-23*04	D2-2	J6*02	CAKDLGYCSSTSCYADRYGMDVW	4
**9/12 week LCL**					
1	V3-23*04	D6-19	J4*02	CARGSSGWW	2
2	V3-23*04	D6-19	J4*02	CARGSSGWW	2
3	V3-23*04	D6-19	J4*02	CARGSSGWW	3
4	V3-23*04	D3-3	J4*02	CAKGCGGVKTIGCDCW	5
5	V3-23*04	D3-10	J6*02	CAKDRGFGELHGMDVW	4
6	V3-23*04	D3-10	J6*02	CAKDRGFGELHGMDVW	7
7	V3-23*04	D3-10	J6*02	CAKDRGFGELHGMDVW	7
8	V3-23*04	D3-10	J6*02	CAKDRGFGELHGMDVW	7
9	V3-23*04	D3-10	J6*02	CAKGRGFGELHGMDVW	7
10	V3-23*04	D3-10	J6*02	CAKDRGFGELHGMDVW	7
11	V3-23*04	D3-10	J6*02	CAKDRGFGELHGMDVW	7
12	V3-23*04	D3-10	J6*02	CAKDRGFGELHGMDVW	7
13	V3-23*04	D3-10	J6*02	CAKDRGFGELHGMDVW	7
14	V3-23*04	D3-10	J6*02	CAKDRGFGELHGMDVW	7
15	V3-23*04	D3-10	J6*02	CAKDRGFGELHGMDVW	7
16	V3-23*04	D3-10	J6*02	CAKDRGFGELHGMDVW	7
17	V3-23*04	D3-10	J6*02	CAKDRGFGELHGMDVW	7
18	V3-23*04	D3-10	J6*02	CAKDRGFGELHGMDVW	7
19	V3-23*04	D3-10	J6*02	CAKDRGFGELHGMDVW	7
20	V3-23*04	D3-10	J6*02	CAKDRGFGELHGMDVW	7
21	V3-23*04	D3-10	J6*02	CAKDRGFGELHGMDVW	7
22	V3-23*04	D3-10	J6*02	CAKDRGFGELHGMDVW	7
23	V3-23*04	D3-10	J6*02	CAKDRGFGELHGMDVW	7
24	V3-23*04	D3-10	J6*02	CAKDRGFGELHGMDVW	7
25	V3-23*04	D3-10	J6*02	CAKDRGFGELHGMDVW	7
26	V3-23*04	D3-10	J6*02	CAKDRGFGELHGMDVW	8
27	V3-23*04	D3-10	J6*02	CAKDRGFGELHGMDVW	8
28	V3-23*04	D3-10	J6*02	CAKDRGFGELHGMDVW	8
29	V3-23*04	D3-10	J6*02	CAKDRGFGELHGMDVW	8
30	V3-23*04	D3-10	J6*02	CAKDRGFGELHGMDVW	8
31	V3-23*04	D2-2	J6*02	CAKDLGYCSSTSCYADRYGMDVW	7

**Figure 7:**
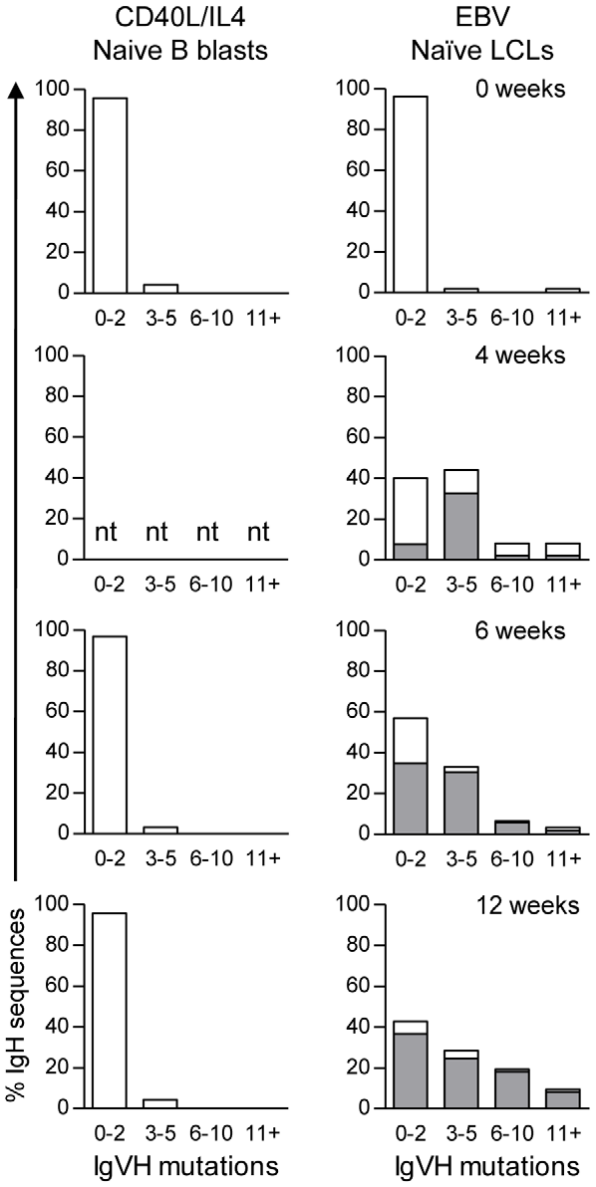
Summary of IgH mutation frequency in mitogen-activated B blasts and LCLs derived from naïve B cells. Histograms show the number of mutations in IgH sequences amplified from CD40L/IL4-stimulated B blasts and EBV-infected LCLs derived from naïve B cell preparations at 0, 4, 6 and 12 week time points. The height of the open bars indicate the percentage of total IgH sequences analysed with a particular frequency of mutations, while the shaded bars indicate the proportion of these IgH sequences detected more than once in each culture i.e. belonging to a CDR3-related clonal family. nt: not tested.

Such EBV-infected bulk cultures were generated from six independent donors, in three cases with a matched CD40L/IL4-stimulated B blast culture. Figure 7 presents a summary of the IgH genotype data from the two types of cultures expressed as histograms; the height of the open bar indicates the percentage of sequences with a particular level of mutation frequency that were amplified from the bulk cultures at different times, while the shaded area of the bar reflects the proportion of those amplified sequences that were members of a clonal family i.e. were detected twice or more. Thus, in starting polyclonal populations, all IgH sequences are unrelated to one another and the great majority are non-mutated. This remains the pattern seen at all times in the CD40L/IL4-expanded B blasts, whereas in EBV-infected cultures, the situation changes with time post-infection. Early on these cultures are also polyclonal and non-mutated but, at around 4 weeks post-infection, mutated IgH sequences appear in significant numbers. From 4 to 12 weeks post-infection, the cultures then become increasingly dominated by a small number of individual clones; in some cases (e.g. Table 3) the most abundant clonotype is non-mutated, while in other cases (e.g. Table 4) it is mutated, much as seen earlier in the limiting dilution culture experiments.

### Mutations within the Ig gene sequences reflect SHM targeting

To ask whether the above Ig sequence changes seen *in vitro* might be products of SHM, we first screened N, NSM and SM subsets pre- and post-infection for expression of AID, an enzyme which is essential (but itself not sufficient) for SHM to occur [39]. In each case, AID transcription was undetectable before infection and was activated by EBV. Figure S2A shows the relevant data from N cell preparations. Interestingly, even though all cells in the culture were actively infected and proliferating by day 7 post-infection, AID levels rose only slowly, not reaching their steady state level until day 35, kinetics that are at least compatible with the delayed appearance of mutated IgH sequences in such cultures. However, we found no correlation between AID expression and Ig gene mutation status. Thus AID transcript levels were similar in N-derived LCLs with and without mutation as well as in NSM- and SM-derived lines; interestingly these AID levels were not only lower than seen in freshly-isolated GC B cells and in 4 Burkitt lymphoma (BL)-derived cell lines, included as SHM-positive controls, but also much lower than those in CD40L/IL4-stimulated B blasts which lack detectable SHM (Figure S2B,C). Moreover N-derived LCLs with and without IgH mutations gave similar results in quantitative RT-PCR assays specific for alternatively-spliced AID mRNAs and for the Polη and UNG co-factors involved in the SHM process [40], [41] (Figure S2C).

**Figure 8:**
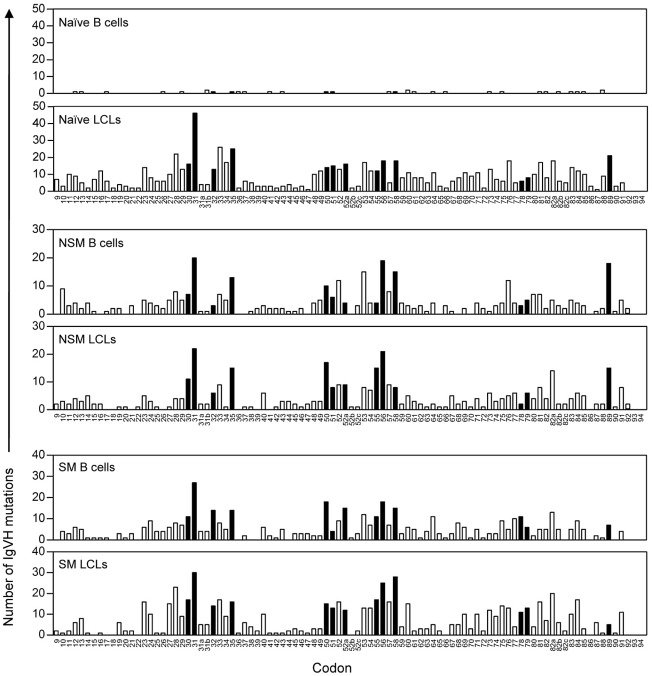
Distribution of IgVH mutations in uninfected B cell subsets and derived LCLs. Histograms show the cumulative pattern of IgVH somatic mutations in naive (N), non-switched memory (NSM) and switched memory (SM) B cell subsets pre-infection and in their derived LCLs. Mutations were scored between codons 9–92 (according to the Kabat numbering system) with codons known to be SHM hotspots shown as filled bars Note that each mutated clonotypic sequence identified within an LCL only contributes once to the overall data.

As a second approach, we asked whether the IgH gene mutations seen in N-derived LCLs had the hallmark of SHM targeting by mapping the location of all IgHV sequence changes seen in pre-infection N, NSM and SM populations and in their derived LCLs. To avoid distortion of the LCL data by numerically dominant clones, here any one mutated clonotypic sequence identified within an LCL only contributes once to the cumulative data. The overall findings are summarised in Figure 8, where the height of the bars indicates the number of times a change in a particular IgHV codon was identified. Sites in the IgHV sequence known to be favoured by the SHM machinery (hot-spots) [42], [43] are identified by filled bars. Focusing first on the data from pre-infection B cell subsets, as already described we found very few mutations in the N-subset, while the NSM and SM subsets showed the expected distribution of mutated sites, with frequent involvement of known SHM hotspots and avoidance of coldspots. Not surprisingly, the NSM- and SM-derived LCLs showed a similar distribution of mutations as in their pre-infection populations. However, it was striking that the same pattern of distribution was also seen among the mutated IgH clonotypes found in N-derived LCLs, at least consistent with these changes being a product of SHM.

Such findings nevertheless leave open the possibility that the mutated clonotypes frequently detected in N-derived LCLs have not arisen from authentic naive cells induced into SHM in vitro but from memory cells already carrying IgH mutations that were present as minor contaminants of the original N cell preparations. Note that the transformation assays on sorted B cell subsets (Figure 2) implied that memory cell contaminants would enjoy no competitive advantage in such a situation. Furthermore, the IgM^+^ IgD^+^ phenotype of the mutated LCLs discounted their being derived from isotype-switched SM cells. However we could not formally discount a contribution from NSM contaminants from the Ig phenotype since both N-derived and NSM-derived transformants would give the same IgM^+^ IgD^+^ LCL signature. The possibility of resolving this issue by genotype was raised by a recent report [44] that a proportion of NSM cells carry mutations in the Bcl6 intronic major mutation cluster (MMC), mutations that (as in conventional memory cells) are thought to arise through SHM mis-targeting during a cell's residence in GCs where Bcl6 is highly expressed [44], [45]. If such mis-targeting is indeed dependent upon active bcl6 transcription, then this would not be expected to occur during EBV-induced B cell transformation in vitro because, as already reported [46] and as we confirmed in the present work (Figure S3), Bcl6 expression is suppressed by growth-transforming EBV infection. We therefore selected 18 LD-LCLs that had a mutated Ig genotype and were derived from N cell preparations, amplified a 718bp region of the Bcl6 MMC, then cloned and sequenced multiple independent amplification products. As internal controls, we included parallel amplifications from sorted naive and memory B cell populations ex vivo. As shown in Table S1, 17/18 Bcl6 MMC sequences amplified from naive B cells were germline, whereas 10/24 sequences from memory cells were mutated; such findings are in close accord with an earlier report [44]. Turning to the Ig-mutated LD-LCLs from N cell infections, we detected both germline and mutated allelic sequences in just 2 lines; the great majority of lines (16/18) only ever yielded germline sequences. Overall, therefore, the bcl6 data are again consistent with the majority of these Ig-mutated LD-LCLs being truly derived from naive cells.

### Intra-clonal diversification of Ig sequences in EBV-transformed bulk cultures of N subset-origin

**Figure 9:**
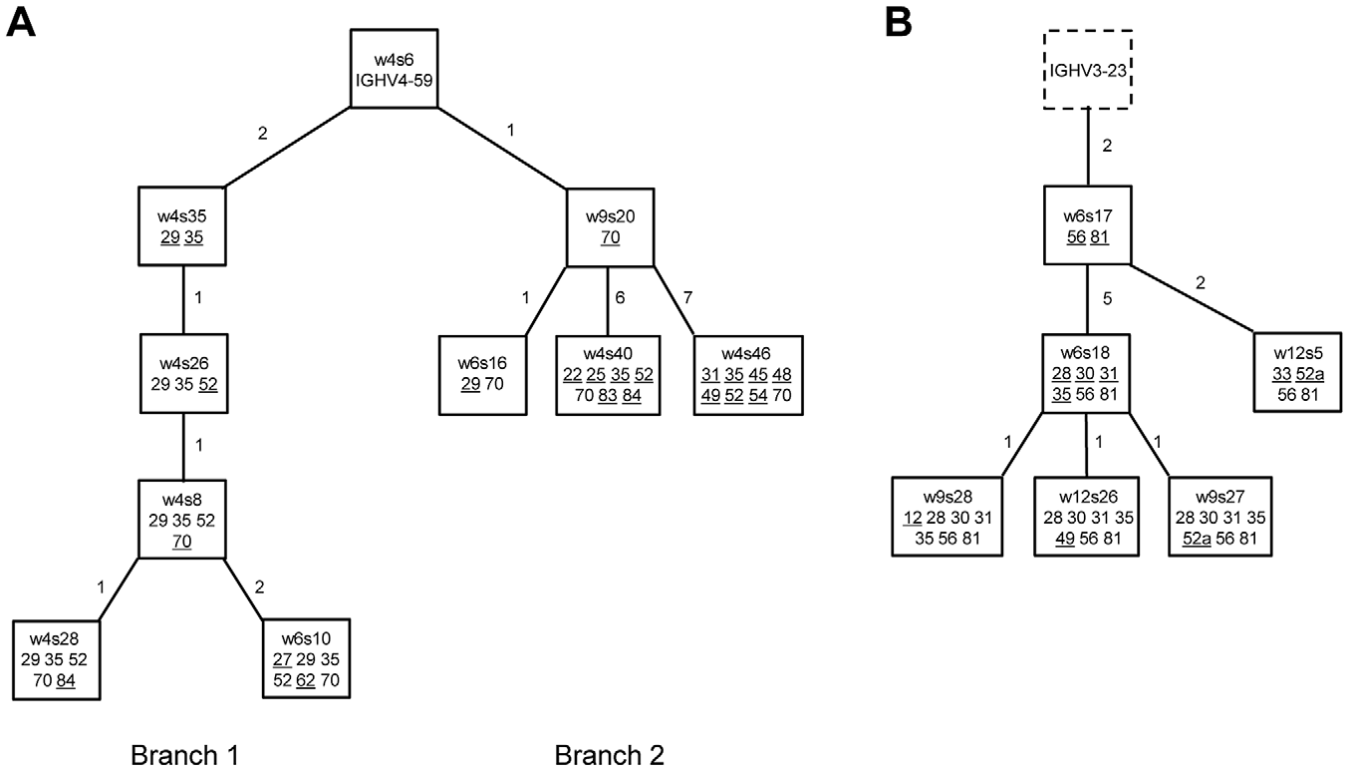
Presumed genealogical trees showing examples of sequence divergence within CDR3-related clones. Each tree is based on clonally-related IgH sequences amplified at different time points from an independent bulk LCL culture derived from EBV-infected naïve B cells. In each case, functional IgH sequences were first aligned with the nearest germline IGH V, D and J alleles to determine the number and location of mutations. Each clonally-related IgH sequence is represented as a box, with the number of incremental mutations shown on each branch; the sequence name and the position of the codon changes (according to the Kabat numbering system) are indicated inside the box while newly acquired mutations are underlined. (A) Prospective analysis of LCL5 identified a sequence (clone w4s6) with zero mutations relative to the nearest germline allele IGHV4-59*01 and a further 9 clonally-related variants (with up to 8 additional changes) which segregated into two major branches. Note that the same mutation at codon 70 was seen in both branch 1 and branch 2 and was therefore assumed to have occurred twice independently. (B) Serial analysis of LCL8 identified a sequence (w6s17) with two mutations relative to the nearest germline allele IGHV3-23*04 and a further 5 clonally-related variants with up to 6 additional sequence changes (note the change in codon 31 involved two substitutions). In this case the nearest germline IGVH allele was a presumed hypothetical intermediate and is therefore represented as a dotted box.

To pursue the question another way, we reasoned that if the mutated clonotypes arising in EBV-infected N cell cultures were truly being generated from naive precursors *in vitro*, then we should be able to detect evidence of intra-clonal sequence diversification within such cultures. In this regard, 28 of the 72 LD-LCLs in which the dominant clonotypic sequence was mutated also contained variants (typically with 1 to 3 nucleotide changes) of that sequence at the single point of harvesting. More informative, however, are the data from bulk N-cell infections sampled over time. Summing data from all 6 experiments, each involving a different N-cell donor, we identified a total of 17 mutated clonotypes that were present on two or more occasions in the same culture between 4 and 12 weeks post-infection; of these, 14/17 clonotypes showed evidence of intra-clonal sequence variation, with a total range of 1–12 sequence changes per clonotype. Two such examples are presented in Figure 9, where in each case the variants can be linked into family trees with different branches arising from a co-resident germline parental sequence. In one case (Figure 9A), we found the parental IgH sequence and 9 clonally-related variants with up to 8 sequence changes; in another case (Figure 9B and Table 4), we found the parental IgH sequence and a further 5 related variants with up to 6 sequence changes. The detailed sequences used to construct these trees are shown in Figures S4A and S4B.

## Discussion

As originally stated, the different views as to how EBV selectively colonises the SM B cell subset *in vivo* hinged on whether this was GC-dependent, involving preferential infection of naïve cells that the virus then induced into memory via GC transit [1], or GC-independent, involving preferential infection/expansion of pre-existing memory cells [2]. Neither view can fully accommodate the more recent finding that EBV also colonises the IgD+ CD27+ NSM subset, not just in healthy carriers [21] but also in patients congenitally devoid of GCs and therefore of SM cells; in such patients the NSM subset is hugely outnumbered by naïve B cells (and is therefore very unlikely to be a preferential target of primary infection) yet it harbours essentially all the latent virus [29]. Given the difficulties posed by such *in vivo* findings, the present work took the reductionist approach of focusing on experimental infection of N, NSM and SM cells *in vitro*. This showed that, while EBV itself does not induce Ig isotype switching, N-derived LCLs remain susceptible to switching induced by surrogate T cell signals. More importantly, in at least a proportion of N cells, EBV infection induces IgH sequence changes which bear the hallmarks of SHM; furthermore, B cell clones with such changes frequently become numerically dominant as the emerging LCL evolves towards monoclonality, typically beginning between 4–6 weeks post-infection. These findings not only suggest alternative routes whereby EBV might become embedded in B cell memory *in vivo* but also strengthen the argument that EBV-induced SHM could contribute to clonal evolution in EBV-associated lymphoproliferative lesions/lymphoma.

It was first necessary to check the susceptibility of the different B cell subsets to EBV infection/transformation. This has been a surprisingly neglected issue after an early study, examining B cell subsets within EBV-infected tonsillar B cell cultures up to 48 hr post-infection, found no differences in the infectability of cells with different surface Ig isotypes [47]. More recently, Dorner et al. also reported that naïve (CD27^−^) and total memory (CD27^+^) B cells from tonsils were equally infectable in short-term assays, although subsequently naïve cells grew slightly quicker and gave slightly better LCL yields from limiting dilution seedings [48]. While naïve (CD27^−^) cells from peripheral blood resembled their tonsillar counterparts, they were more infectable than peripheral blood memory (CD27^+^) preparations; this could not be explained at the level of receptor (CD21)/co-receptor (HLAII) expression but was ascribed to heterogeneity among circulating memory cells in expression of another putative co-receptor, α5β1 integrin [49]. Comparisons with the present work are difficult because Dorner et al. employed an unusual recombinant EBV strain (lacking one of the latent proteins, LMP2A) whose low titre preparations necessitated the use of spinoculation to achieve measurable rates of infection. The present work, using wild-type EBV and conventional infection protocols, did not detect any significant difference in virus binding, infectability or transformability under limiting conditions between peripheral blood N, NSM and SM preparations (Figure 2). Likewise, another recent report comparing naïve (CD27^−^) and total memory (CD27^+^) B cells from blood also found no significant difference in transformability [50]. Interestingly we noticed that, in contrast to the ease with which LCLs grow out from positive wells at the high end of transformation assays, only a subset of positive wells arising under limiting conditions could be expanded to establish LCLs. This likely reflects the fact, recently noted by others [51], [52] and also apparent from our CDR3 spectratyping of EBV-infected bulk cultures (Figure 6), that the process of LCL establishment is associated with significant clonal selection, a hurdle which cultures with small seed populations may fail to overcome. The basis of this selection remains to be determined. However, from the point of view of the present work, we can conclude that N, NSM and SM populations show similar levels of attrition at this stage.

With respect to virus-induced changes in cellular phenotype, as many have observed [52]–[56], EBV-transformed LCLs converge on a similar “lymphoblastoid” phenotype, irrespective of the precise differentiation stage of the target B cell; importantly, that phenotype includes the memory marker CD27, which is induced on N-derived LCLs to levels similar to those retained on memory LCLs [31]. By contrast, EBV transformation does not lead to convergence of Ig isotype expression. Thus we detected no Ig isotype switching in N- or NSM-derived LCLs, whether analysed by transcript-specific RT/PCR assay or by protein expression (Figure 3A, B and Table 1). This accords with early work on the IgM^+^ IgD^+^ cell fraction from peripheral blood where EBV infection induced IgM but not IgG or IgA production [57]. However it apparently contradicts another study [58] in which switch circles, considered an early marker of class switching, and some switch transcripts were detected in naïve B cells after EBV infection *in vitro*. In that same study, however, evidence of isotype switching at the protein level was only given for clones of the EBV-negative BL line, Ramos, that had been transfected to express LMP1 [58], the EBV latent cycle protein that mimics many of the effects of CD40 ligation [59], [60]. Likewise another study [61] linking EBV with the induction of switch recombinase activity was based on viral infection of a B lymphoma line BJAB or on EBV-positive BL cells. In neither study, therefore, was there definitive evidence of EBV-induced isotype switching in the setting of normal B cells; indeed others have used EBV-infected B cells as isotype-stable substrates in order to study switching induced by exposure to cytokines and/or CD40 ligation [62], [63]. We indeed confirmed that EBV transformation still leaves early passage N- and NSM-derived LCLs responsive to exogenous signals, CD40L/IL4/IL21, that mimic T cell-derived switch signals *in vivo* (Figure 3C). Thus EBV infection itself can induce naïve B cells to acquire an NSM-like surface phenotype (IgD^+^ CD27^+^) and, with appropriate T cell help, a SM-like phenotype (IgD^−^ CD27^+^).

Key findings arose when the study turned to virus-induced changes Ig genotype. In limiting dilution seeding experiments, designed to generate transformed populations of limited clonality, we were surprised to find that >30% N-derived LD-LCLs contained mutated IgH sequences, a result seen consistently across experiments on 8 different B cell donors (Figure 4). Combining data from all these mutated clonotypes gives a mean of 9.2 nucleotide substitutions per IgH sequence, a value substantially higher than the background that would expected from PCR amplification error and in stark contrast to the mean values of 0.4 substitutions per sequence seen in the original N cell-sorts and of 0.5 substitutions per sequence seen in CD40L/IL4-expanded N cell cultures. Interestingly the 9.2 value is below the mean number of mutations (15.2) we observed in SM cell preparations but similar to the mean (7.2) seen in NSM cells and their derived LCLs. This similarity, and the fact that all N-derived transformants were IgM+ IgD+, meant that if such mutated clonotypes were arising from pre-existing memory cells in the original N cell sorts, then the source of contamination must be NSM cells. However, such a possibility seems at odds both with the high purity of N cell preparations, shown to be >99% by IgD/CD27 staining and 97% by Ig gene sequencing (Figure 1), and with the fact that NSM cells were not more transformable than N cells when compared in parallel assays (Figure 2) nor more likely to survive the clonal selection that then occurs with transition from transformed cell focus to established LCL. Having said that, we cannot entirely discount the possibility that some of these mutated clonotypes derive from NSM cells contaminating the N cell sorts, particularly in the rare cases of clones that also carry bcl6 mutations. Were NSM cells to be the source of even a small fraction of the Ig-mutated LCLs detected in the present work, this would be worthy of further attention since it implies that NSM-derived transformants enjoy an advantage over EBV-infected naive cells that is only apparent when the two are competing in mixed culture.

However we would argue that this is not the main explanation for our findings. Rather, the evidence suggests that many of the mutated IgH clonotypes seen in LCLs derived from N cell preparations have arisen *in vitro* from naïve precursor cells. This rests on the observation of ongoing intra-clonal diversification within a culture. Early in the work we noted that clonotypic sequences varying by 1–3 nucleotides could be amplified from some LD-LCLs, but such examples, coming from single harvests made up to 12 weeks post-infection, are difficult to interpret in isolation. We therefore set up EBV-infected bulk N cell cultures that could be harvested prospectively, with parallel cultures of CD40L/IL4-stimulated N cell blasts as controls. Such experiments, carried out on N cell preparations from 6 different donors, led to the following conclusions. Firstly, the EBV-infected populations remain polyclonal for 4 weeks but thereafter move quite rapidly to oligo- or mono-clonality (N.B. a previous CDR3 spectratyping study also reported clonal dominance within 12 weeks [52]); by contrast parallel cultures of CD40L/IL4-stimulated blasts remain entirely polyclonal despite extensive proliferation (Figure 6, Tables 3, 4). Secondly, mutated IgH genotypes regularly appear in EBV-infected N cell cultures (but only rarely in the CD40L/IL4 cultures) and, in 5 of 6 experiments, grew out to become either the dominant or significant sub-dominant clones. Thirdly, and most importantly, in some cases family trees could be drawn tracing intra-clonal diversification with time in the emerging LCL (Figure 9). To date, almost all other reports of intra-clonal diversification in an LCL have come from one late passage, monoclonal antibody-producing line studied long after its establishment [64]–[66]; interestingly, however, a recent study [31] found that 1 of 3 naïve B cell-derived limiting dilution LCLs (included as controls in a study of GC cell transformants) showed significant levels of IgH gene diversification, a result which here we show to be representative of a general trend.

It is striking that the IgH mutations observed in this study are frequently situated at known hotspots of SHM targeting (Figure 8). This, and the known ability of EBV to induce AID and other factors involved in the SHM process [58], [64], [67], leads us to suggest that the changes are indeed AID-mediated. However we cannot say why EBV-induced, AID-driven IgH gene mutation was detected in only a proportion of N-derived LCLs in these experiments, when the expression of AID and related co-factors UNG and DNA polη was equally well induced in all lines (Figure S2). Mutation may be a stochastic event or possibly dependent on the cellular context, i.e. there may be differences among naïve B cells in their inducibility to IgH gene mutation, just as naive populations are heterogeneous in their response to isotype-switch signals *in vitro* [35].

These findings potentially bear upon two key aspects of EBV biology *in vivo*. The first concerns the means whereby the virus sequesters in memory B cell populations. It is clear from Ig genotyping of infected cells both in the transient lymphoproliferations seen during primary infection in IM tonsils [6] and in the progressive lymphoproliferative lesions arising in acutely immunosuppressed transplant recipients [10], that at least some naive B cells do become infected *in vivo*. Hence the subsequent absence of the virus from naive populations in the immunocompetent host must reflect either clearance of these EBV-infected naive B cells by the T cell response (which could happen were they unable to make the transition to an antigen-negative resting state) or the cell's acquisition of a memory Ig genotype/phenotype. Our data raise the possibility that EBV infection *per se* could induce an NSM Ig genotype/phenotype without germinal centre transit, while T cell signals, perhaps in the extra-follicular environment [5], [21] could induce switching to SM status.

The second aspect of EBV biology touched on by this work concerns the mechanisms whereby virus infection contributes to B-lymphomagenesis. It is increasingly clear that the relatively low efficiency with which a B cell, driven into the first cell cycle by EBV infection, achieves outgrowth to an LCL (1–10% by some calculations [68]), cannot be fully ascribed to the inherent limitations of *in vitro* culture. Successful outgrowth reportedly requires escape from the oncogenic stress and attendant DNA damage response to which hyper-proliferating cells are subject within the first week (i.e. first 5–6 divisions) post-infection [69] and also survival through a transient period of genetic instability peaking around 4 weeks post-infection [51]. It remains to be seen to what extent virus-driven SHM activation contributes to these phenomena. However our findings on clonal evolution in N cell cultures from 4–8 weeks post-infection would suggest that naive cells in which SHM has targeted the Ig locus have a competitive edge in outgrowth to form an LCL. One attractive possibility is that the competitive advantage comes not from Ig mutation *per se*, but from mutations in other growth/survival-promoting genes that are coincidentally targeted by the SHM machinery. If such a mechanism, thought to be involved in the genesis of many lymphomas of GC/post-GC origin [70]–[72] plays some role in determining clonal selection during LCL outgrowth, then it represents a second way, in addition to expression of EBV's growth-transforming latent genes, whereby the virus may contribute to lymphomagenesis.

## Materials and Methods

### Ethics statement

These studies were approved by the University of Birmingham and the South Birmingham Research Ethics Committee, UK (07/H/1207/271). All blood donors provided written informed consent.

### B cell isolation and purification of B cell subsets

Peripheral blood mononuclear cells (PBMC) were isolated by Ficoll-Isopaque density centrifugation of adult buffy coat samples (National Blood Service, UK) and B cells positively selected by immunomagnetic cell isolation using CD19 Pan B Dynabeads (Life Technologies) followed by bead detachment. B cell purity was assessed by staining with PE-Cy5-conjugated mouse anti-human CD20 (1∶40, Dako) monoclonal antibody (mAb) and FACS analysis on a Coulter Epics XL-MCL flow cytometer. To isolate B cell subsets, purified B cells were co-stained with FITC-labelled anti-IgD (1∶40, Dako) and PE-labelled anti-CD27 (1∶20, BD Pharmingen) antibodies (Abs). IgD^+^ CD27^−^ (naïve), IgD^−^ CD27^+^ (switched memory) and IgD^+^ CD27^+^ (non-switched memory) B cell subsets were simultaneously collected during a single FACS sorting procedure on a MoFlo sorter (Beckman Coulter). Small aliquots of sorted cells were used to re-analyse purity of subsets thus isolated.

### Virus binding assays

Expression of the EBV receptor, CD21 [73], [74], was assessed by FACS analysis after staining B cells and subsets with PE-Cy5 mouse anti-human CD21 mAb (1∶40, BD Pharmingen). For virus binding assays, 2×10^5^ B cells from each subset were incubated with B95.8 strain EBV at 100 moi (100 EBV genome copies/cell) for 3 hours at 4°C and the number of cell-bound virus genomes determined by real time quantitative PCR as described [75].

### In vitro transformation assays and establishment of LCLs

The transformation efficiency of different B cell subsets was assayed by two different experimental assays. In one assay, 1×10^6^ N, NSM and SM B cells were exposed to a standard virus dose (50 moi) for 1 hour at 37°C before seeding at two-fold limiting dilutions (from 1000 cells/well) into replicate wells of a 96-well plate containing human fibroblast feeder cells. In the second assay, 2×10^5^ N and NSM cells were exposed for 1 hour at 37°C to two-fold virus dilutions (from 50 moi) before seeding 1000 cells into replicate wells of a 96-well plate containing human fibroblast feeder cells. In both cases, cultures were maintained in RPMI 1640 medium supplemented with 2 mM glutamine and 10% v/v FCS for 6 weeks at which time wells with typical foci of EBV-transformed lymphoblastoid cells were scored positive. At that time, all positive cultures at the end-point of the titration (i.e. conditions where only a fraction of replicate wells had transformed) were split, initially into duplicate microtest wells, then the duplicates pooled into a 1 ml well and thence into a 5 ml culture, in order to establish limiting dilution (LD) LCLs for analysis. The proportion of cultures which could be successfully expanded in this way over the following 4–6 weeks was recorded; as an internal control, when positive cultures from the high end of the titration (i.e. conditions giving 100% positive wells) were likewise treated, all could be successfully expanded to LCLs. In other experiments, bulk LCLs of N, NSM and SM origin were generated by infecting 2×10^6^ cells of each type with a standard virus dose (50 moi), seeding into 2 ml wells and then expanding into a 25 ml flask; flask cultures were then maintained in exponential growth by passaging twice weekly.

### Generation of mitogen-activated B blasts

Activated B blasts were generated by culturing naive B cell preparations with irradiated CD40L-expressing mouse L cells in the presence of IL-4 [76]. Irradiated L cells were added to 24-well tissue culture plates (0.2×10^6^ cell/well) and allowed to adhere over night before the addition of 0.5×10^6^ B cells in 3 ml CD40L blast medium (Iscove's Medium supplemented with 10% human serum and 50 ng/ml IL-4) . Cultures were passaged onto fresh L cells twice weekly and maintained for up to 12 weeks.

### Cytokine stimulation of Ig isotype class switching

Ig isotype switching was induced by culturing N and NSM B cell-derived LCLs with irradiated CD40L-expressing mouse L cells in the presence of IL4 and IL21 [35]. Irradiated L cells were added to 12-well tissue culture plates (0.2×10^6^ cell/well) per well prior to the addition of 2×10^6^ LCL cells in 1.5 ml CD40L blast medium supplemented with 50 ng/ml IL-4 and 50 ng/ml IL21. The cells were split after 3–4 days in culture, and harvested after 7 days for Ig staining.

### Antibody staining for surface markers and Ig isotypes

LCLs were stained with fluorochrome-labelled mAbs against CD23 (Immunotech) and CD27 (BD Pharmingen) to determine the expression of these surface markers. Cells were also analysed for surface Ig by staining with fluorochrome-labelled isotype-specific mAbs to IgD, IgM, IgG and IgA (Dako). All mAbs were used at 1∶20 and staining analysed using a Coulter Epics XL-MCL flow cytometer and WinMDI software.

### RT/PCR assay for Ig isotype-specific transcripts

Total RNA was extracted from 2×10^6^ cells using a Nucleospin II RNA isolation kit (Macherey Nagel) and cDNA was synthesised using random hexamers and AMV-RT (Roche). Ig heavy chain (IgH) sequences were PCR-amplified using a single consensus forward primer FR1c within framework region (FR)1 [77] and one of four IgH constant region reverse primers specific for IgM, IgD, IgG or IgA [78]. Each PCR amplification contained 0.8 µM FR1c primer, 0.8 µM IgH family-specific reverse primer, 200 µM dNTPs, 1.5 mM MgCl_2_ and 5units Red Hot DNA Polymerase (ABgene). The first PCR cycle consisted of a denaturing step of 94°C for 5 mins, followed by 35 cycles of denaturation at 94°C for 30 s, annealing at 61°C for 60 s and extension at 72°C for 60 s (10 mins in the last cycle). PCR products were run on a 1.5% agarose gel and IgH products (600 bp) identified by ethidium bromide staining.

### PCR amplification and analysis of IgH sequences

DNA was extracted from up to 2×10^6^ cells using a DNeasy Tissue kit (Qiagen). Ig heavy chain variable (IgHV) sequences spanning FR1, CDR1, FR2, CDR2, FR3, and CDR3 were PCR-amplified using a single consensus forward primer FR1c 5′AGGTGCAGCTGSWGSAGTCDGG3′ and a mixture of JH family-specific reverse primers JH1/2/4/5 5′ACCTGAGGAGACGGTGACCAGGGT3′, JH3 5′TACCTGAAGAGACGGTGACCATTGT3′ and JH6 5′ACCTGAGGAGACGGTGACCGTGGT3′ described previously [10], [77]. PCR amplification was performed using the Expand High Fidelity PCR system (Roche) in a reaction containing 1 µg heat denatured genomic DNA, 0.8 µM FR1c primer, 0.26 µM each JH primer, 200 µM each dNTP, 1.5 mM MgCl_2_ and 3.5units Expand High Fidelity DNA polymerase. The first PCR cycle consisted of a denaturing step of 94°C for 5 mins, followed by 30 cycles of denaturation at 94°C for 30 s, annealing at 61°C for 60 s and extension at 72°C for 60 s (10 mins in the last cycle). PCR products were then separated by electrophoresis on a 8% polyacrylamide gel, and bands corresponding to the approximately 350 bp IgH products excised and the DNA eluted. Finally each purified PCR product was cloned using the pGEM-T Easy Vector System (Promega), and following bacterial transformation, 3–5 independent bacterial clones were subjected to DNA sequencing on a Applied Biosystems ABI3730 DNA analyser (Functional Genomics, University of Birmingham).

IgH sequences were analysed using the V-Quest (http://www.imgt.org) [79] and VBase (http://vbase.mrc-cpe.cam.ac.uk) directories to identify the nearest IGHV, IGHD and IGHJ germline alleles; at this point sequences carrying out-of-frame CDR3 junction regions or chimaeric sequences generated by PCR crossover between two different IgH alleles were discarded from the analysis. Translated CDR3 junction sequences were generated using V-Quest. IgH sequences carrying the same IGHV allele and identical translated CDR3 sequences were assigned to the same CDR3 clone. IgH sequences with 0, 1 or 2 nucleotide changes in the IGHV region (between codons 9–92, based on the Kabat numbering system [80]) were considered to be naive, and those showing more than 2 nucleotide changes were considered to be mutated [10]. Sequence alignments were generated and visualised using BioEdit (http://www.mbio.ncsu.edu/bioedit/bioedit.html).

### Taqman RT-PCR assay for AID transcripts

Total RNA isolation and cDNA synthesis reactions were carried out as described above. AID, uracil N-glycosylase (UNG) and DNA polymerase η transcripts were quantified by real time PCR on an ABI Prism 7500 Sequence Detection System using commercially available reagents (Applied Biosystems). Values were normalised against GAPDH mRNA levels in the same cells and then expressed relative to that seen in a reference AID-positive B cell line, Ramos-BL [81]. A primer/probe combination to specifically detect an AID splice variant lacking exon 4 was designed using Primer Express software (Applied Biosystems).

### Immunoblotting

Western blotting was carried out as described previously [82] using mAbs to: EBNA1 (1H4), EBNA2 (PE2), LMP1 (CS1-4), BZLF1 (BZ-1). Bcl6 was detected using a rabbit polyclonal Ab (N3, Santa Cruz).

### Bcl6 mutation analysis

A 765 bp fragment of the Bcl6 MMC was PCR-amplified with the primers 5′ CAAATGCTTTGGCTCCAAGTTTTCT 3′ and 5′ AGGAAGATCACGGCTCTGAAAGG 3′ PCR amplification using the Expand High Fidelity PCR system (Roche) in a reaction containing 1 µg heat denatured genomic DNA, 1 µM each primer, 200 µM each dNTP, 1.5 mM MgCl_2_ and 3.5 units Expand High Fidelity DNA polymerase. The first PCR cycle consisted of a denaturing step of 94°C for 5 mins, followed by 40 cycles of denaturation at 94°C for 30 s, annealing at 60°C for 60 s and extension at 72°C for 60 s (10 mins in the last cycle). PCR products were then separated by electrophoresis on a 1.5% agarose gel, the appropriate bands excised and the DNA eluted. Purified PCR products were cloned using the pGEM-T Easy Vector System (Promega), and following bacterial transformation, 3–5 independent bacterial clones were subjected to DNA sequencing. A 716 bp region of each amplified Bcl6 sequence was aligned to a Bcl6 reference sequence using BioEdit.

## Supporting Information

Figure S1 **IgH sequences from 3 limiting dilution LCLs established from LCL6.** In each case the amplified IgH sequence is aligned with the nearest germline IGHV, IGHD and IGHJ alleles, with sequence identities shown as dots. (A) shows an example of a germline IgH sequence while (B) and (C) show examples of mutated IgH sequences with 5 and 7 changes, respectively, taken from Table 2. Complementarity determining regions CDR1, CDR2 and CDR3 are shaded. Note that the IgVH sequence starts at codon 9 and nucleotide changes in CDR3 are ignored.
(TIF)

Figure S2 **Expression of AID and accessory factors involved in SHM.** (A) AID expression was quantified by real time RT-PCR in a bulk LCL culture at various time points post infection. Normalised AID values are expressed relative to the reference BL line Ramos. (B) AID expression quantified by real time RT-PCR in uninfected naïve (N), non-switched memory (NSM) and switched memory (SM) B cells and N-, NSM- and SM-derived limiting dilution LCLs. Data are shown separately for N-derived LCLs with germline or mutated IgH sequences. Two T cell lines (Jurkat and Molt4) and two pre-B cell lines (Nalm6 and Nalm16) were included as negative controls, while four BL lines (Akata-BL, P3HR1-BL, Rael-BL and Ramos-BL) and two germinal centre (GC) B cell preparations served as positive controls. Note that the reference Ramos-BL cell line has the lowest AID expression of the four BL lines. AID values are expressed as in panel A. (C) Expression of full length (FL) AID transcripts, exon4-deleted AID variant transcripts, UNG transcripts and DNA polη transcripts measured by real time RT-PCR. Data are shown from 5 representative naive B cell-derived limiting dilution LCL cultures with either germline or mutated IgH genotypes and 2 polyclonal CD40L/IL4 stimulated N cell-derived B blasts (Blast 1 and Blast 2) from separate donors. Normalised values are expressed relative to Ramos-BL (AID) or Jurkat cells (UNG and DNA polη). Data are the mean (+/− SD) of triplicate readings.
(TIF)

Figure S3 **Bcl6 expression is downregulated following EBV transformation.** Immunoblots show expression of Bcl6 in representative LD-LCL cultures carrying either germline or mutated IgH genotypes. Two EBV-positive BL cell lines (Wan-BL, Rael-BL) were included as positive controls for Bcl6 expression. Calregulin was used as a loading control.
(TIF)

Figure S4 **Examples of clonal variation within LCL bulk cultures.** Shown are selected clonally-related IgH sequences amplified from naive B cell derived bulk LCL cultures which were used to construct the genealogical trees shown in Figure 8. The sequence name reflects the time post infection (in weeks, w) at which the clone was isolated and the sequence number (s), while the number in parentheses indicates the number of mutations relative to the nearest germline sequence shown on the top line. Sequence identities are shown as dots; complementarity determining regions CDR1, CDR2 and CDR3 are shaded. (A) shows the results for LCL5 while (B) shows the results for LCL8.
(TIF)

Table S1 **Bcl6 MMC mutation status in naive B cell-derived LD LCL clones with mutated IgH genotypes.**
(DOC)

## References

[1] Thorley-Lawson DA (2001). Epstein-Barr virus: exploiting the immune system.. Nat Rev Immunol.

[2] Kuppers R (2003). B cells under influence: transformation of B cells by Epstein-Barr virus.. Nat Rev Immunol.

[3] Rickinson AB, Kieff E, Knipe DM, Howley PM (2006). Epstein-Barr Virus.. Fields Virology. 5 ed.

[4] Niedobitek G, Agathanggelou A, Herbst H, Whitehead L, Wright DH (1997). Epstein-Barr virus (EBV) infection in infectious mononucleosis: virus latency, replication and phenotype of EBV-infected cells.. J Pathol.

[5] Kurth J, Spieker T, Wustrow J, Strickler GJ, Hansmann LM (2000). EBV-infected B cells in infectious mononucleosis: viral strategies for spreading in the B cell compartment and establishing latency.. Immunity.

[6] Kurth J, Hansmann ML, Rajewsky K, Kuppers R (2003). Epstein-Barr virus-infected B cells expanding in germinal centers of infectious mononucleosis patients do not participate in the germinal center reaction.. Proc Natl Acad Sci U S A.

[7] Babcock GJ, Decker LL, Volk M, Thorley-Lawson DA (1998). EBV persistence in memory B cells in vivo.. Immunity.

[8] Joseph AM, Babcock GJ, Thorley-Lawson DA (2000). EBV persistence involves strict selection of latently infected B cells.. J Immunol.

[9] Souza TA, Stollar BD, Sullivan JL, Luzuriaga K, Thorley-Lawson DA (2005). Peripheral B cells latently infected with Epstein-Barr virus display molecular hallmarks of classical antigen-selected memory B cells.. Proc Natl Acad Sci U S A.

[10] Timms JM, Bell A, Flavell JR, Murray PG, Rickinson AB (2003). Target cells of Epstein-Barr-virus (EBV)-positive post-transplant lymphoproliferative disease: similarities to EBV-positive Hodgkin's lymphoma.. Lancet.

[11] Brauninger A, Spieker T, Mottok A, Baur AS, Kuppers R (2003). Epstein-Barr virus (EBV)-positive lymphoproliferations in post-transplant patients show immunoglobulin V gene mutation patterns suggesting interference of EBV with normal B cell differentiation processes.. Eur J Immunol.

[12] Capello D, Cerri M, Muti G, Berra E, Oreste P (2003). Molecular histogenesis of posttransplantation lymphoproliferative disorders.. Blood.

[13] Capello D, Cerri M, Muti G, Lucioni M, Oreste P (2006). Analysis of immunoglobulin heavy and light chain variable genes in post-transplant lymphoproliferative disorders.. Hematol Oncol.

[14] Vakiani E, Basso K, Klein U, Mansukhani MM, Narayan G (2008). Genetic and phenotypic analysis of B-cell post-transplant lymphoproliferative disorders provides insights into disease biology.. Hematol Oncol.

[15] Rajewsky K (1996). Clonal selection and learning in the antibody system.. Nature.

[16] Muramatsu M, Kinoshita K, Fagarasan S, Yamada S, Shinkai Y (2000). Class switch recombination and hypermutation require activation-induced cytidine deaminase (AID), a potential RNA editing enzyme.. Cell.

[17] Revy P, Muto T, Levy Y, Geissmann F, Plebani A (2000). Activation-induced cytidine deaminase (AID) deficiency causes the autosomal recessive form of the Hyper-IgM syndrome (HIGM2).. Cell.

[18] Ta VT, Nagaoka H, Catalan N, Durandy A, Fischer A (2003). AID mutant analyses indicate requirement for class-switch-specific cofactors.. Nat Immunol.

[19] Barreto V, Reina-San-Martin B, Ramiro AR, McBride KM, Nussenzweig MC (2003). C-terminal deletion of AID uncouples class switch recombination from somatic hypermutation and gene conversion.. Mol Cell.

[20] Klein U, Rajewsky K, Kuppers R (1998). Human immunoglobulin (Ig)M+IgD+ peripheral blood B cells expressing the CD27 cell surface antigen carry somatically mutated variable region genes: CD27 as a general marker for somatically mutated (memory) B cells.. J Exp Med.

[21] Chaganti S, Heath EM, Bergler W, Kuo M, Buettner M (2009). Epstein-Barr virus colonization of tonsillar and peripheral blood B-cell subsets in primary infection and persistence.. Blood.

[22] Agematsu K, Nagumo H, Shinozaki K, Hokibara S, Yasui K (1998). Absence of IgD-CD27(+) memory B cell population in X-linked hyper-IgM syndrome.. J Clin Invest.

[23] Weller S, Faili A, Garcia C, Braun MC, Le Deist FF (2001). CD40-CD40L independent Ig gene hypermutation suggests a second B cell diversification pathway in humans.. Proc Natl Acad Sci U S A.

[24] Ma CS, Pittaluga S, Avery DT, Hare NJ, Maric I (2006). Selective generation of functional somatically mutated IgM+CD27+, but not Ig isotype-switched, memory B cells in X-linked lymphoproliferative disease.. J Clin Invest.

[25] Klein U, Kuppers R, Rajewsky K (1997). Evidence for a large compartment of IgM-expressing memory B cells in humans.. Blood.

[26] van Es JH, Meyling FH, Logtenberg T (1992). High frequency of somatically mutated IgM molecules in the human adult blood B cell repertoire.. Eur J Immunol.

[27] Huang C, Stewart AK, Schwartz RS, Stollar BD (1992). Immunoglobulin heavy chain gene expression in peripheral blood B lymphocytes.. J Clin Invest.

[28] Tangye SG, Good KL (2007). Human IgM+CD27+ B cells: memory B cells or “memory” B cells?. J Immunol.

[29] Chaganti S, Ma CS, Bell AI, Croom-Carter D, Hislop AD (2008). Epstein-Barr virus persistence in the absence of conventional memory B cells: IgM+IgD+ CD27+ B cells harbour the virus in X-linked lymphoproliferative disease patients.. Blood.

[30] Conacher M, Callard R, McAulay K, Chapel H, Webster D (2005). Epstein-Barr virus can establish infection in the absence of a classical memory B-cell population.. J Virol.

[31] Siemer D, Kurth J, Lang S, Lehnerdt G, Stanelle J (2008). EBV transformation overrides gene expression patterns of B cell differentiation stages.. Mol Immunol.

[32] Fujieda S, Zhang K, Saxon A (1995). IL-4 plus CD40 monoclonal antibody induces human B cells gamma subclass-specific isotype switch: switching to gamma 1, gamma 3, and gamma 4, but not gamma 2.. J Immunol.

[33] Gascan H, Gauchat JF, Aversa G, Van Vlasselaer P, de Vries JE (1991). Anti-CD40 monoclonal antibodies or CD4+ T cell clones and IL-4 induce IgG4 and IgE switching in purified human B cells via different signaling pathways.. J Immunol.

[34] Splawski JB, Fu SM, Lipsky PE (1993). Immunoregulatory role of CD40 in human B cell differentiation.. J Immunol.

[35] Avery DT, Bryant VL, Ma CS, de Waal Malefyt R, Tangye SG (2008). IL-21-induced isotype switching to IgG and IgA by human naive B cells is differentially regulated by IL-4.. J Immunol.

[36] Galibert L, van Dooren J, Durand I, Rousset F, Jefferis R (1995). Anti-CD40 plus interleukin-4-activated human naive B cell lines express unmutated immunoglobulin genes with intraclonal heavy chain isotype variability.. Eur J Immunol.

[37] Fecteau JF, Neron S (2003). CD40 stimulation of human peripheral B lymphocytes: distinct response from naive and memory cells.. J Immunol.

[38] Razanajaona D, Denepoux S, Blanchard D, de Bouteiller O, Liu YJ (1997). In vitro triggering of somatic mutation in human naive B cells.. J Immunol.

[39] de Yebenes VG, Ramiro AR (2006). Activation-induced deaminase: light and dark sides.. Trends Mol Med.

[40] Wu X, Darce JR, Chang SK, Nowakowski GS, Jelinek DF (2008). Alternative splicing regulates activation-induced cytidine deaminase (AID): implications for suppression of AID mutagenic activity in normal and malignant B cells.. Blood.

[41] Weill JC, Reynaud CA (2008). DNA polymerases in adaptive immunity.. Nat Rev Immunol.

[42] Dorner T, Foster SJ, Brezinschek HP, Lipsky PE (1998). Analysis of the targeting of the hypermutational machinery and the impact of subsequent selection on the distribution of nucleotide changes in human VHDJH rearrangements.. Immunol Rev.

[43] Rogozin IB, Diaz M (2004). Cutting edge: DGYW/WRCH is a better predictor of mutability at G:C bases in Ig hypermutation than the widely accepted RGYW/WRCY motif and probably reflects a two-step activation-induced cytidine deaminase-triggered process.. J Immunol.

[44] Seifert M, Kuppers R (2009). Molecular footprints of a germinal center derivation of human IgM+(IgD+)CD27+ B cells and the dynamics of memory B cell generation.. J Exp Med.

[45] Pasqualucci L, Migliazza A, Fracchiolla N, William C, Neri A (1998). BCL-6 mutations in normal germinal center B cells: evidence of somatic hypermutation acting outside Ig loci.. Proc Natl Acad Sci U S A.

[46] Carbone A, Gaidano G, Gloghini A, Pastore C, Saglio G (1997). BCL-6 protein expression in AIDS-related non-Hodgkin's lymphomas: inverse relationship with Epstein-Barr virus-encoded latent membrane protein-1 expression.. Am J Pathol.

[47] Ehlin-Henriksson B, Gordon J, Klein G (2003). B-lymphocyte subpopulations are equally susceptible to Epstein-Barr virus infection, irrespective of immunoglobulin isotype expression.. Immunology.

[48] Dorner M, Zucol F, Berger C, Byland R, Melroe GT (2008). Distinct ex vivo susceptibility of B-cell subsets to epstein-barr virus infection according to differentiation status and tissue origin.. J Virol.

[49] Dorner M, Zucol F, Alessi D, Haerle SK, Bossart W (2010). beta1 integrin expression increases susceptibility of memory B cells to Epstein-Barr virus infection.. J Virol.

[50] Iskra S, Kalla M, Delecluse HJ, Hammerschmidt W, Moosmann A (2010). Toll-like receptor agonists synergistically increase proliferation and activation of B cells by epstein-barr virus.. J Virol.

[51] Lacoste S, Wiechec E, Dos Santos Silva AG, Guffei A, Williams G (2010). Chromosomal rearrangements after ex vivo Epstein-Barr virus (EBV) infection of human B cells.. Oncogene.

[52] Ryan JL, Kaufmann WK, Raab-Traub N, Oglesbee SE, Carey LA (2006). Clonal evolution of lymphoblastoid cell lines.. Lab Invest.

[53] Gregory CD, Kirchgens C, Edwards CF, Young LS, Rowe M (1987). Epstein-Barr virus-transformed human precursor B cell lines: altered growth phenotype of lines with germ-line or rearranged but nonexpressed heavy chain genes.. Eur J Immunol.

[54] Chaganti S, Bell AI, Pastor NB, Milner AE, Drayson M (2005). Epstein-Barr virus infection in vitro can rescue germinal center B cells with inactivated immunoglobulin genes.. Blood.

[55] Mancao C, Altmann M, Jungnickel B, Hammerschmidt W (2005). Rescue of “crippled” germinal center B cells from apoptosis by Epstein-Barr virus.. Blood.

[56] Bechtel D, Kurth J, Unkel C, Kuppers R (2005). Transformation of BCR-deficient germinal-center B cells by EBV supports a major role of the virus in the pathogenesis of Hodgkin and posttransplantation lymphomas.. Blood.

[57] Miyawaki T, Butler JL, Radbruch A, Gartland GL, Cooper MD (1991). Isotype commitment of human B cells that are transformed by Epstein-Barr virus.. Eur J Immunol.

[58] He B, Raab-Traub N, Casali P, Cerutti A (2003). EBV-encoded latent membrane protein 1 cooperates with BAFF/BLyS and APRIL to induce T cell-independent Ig heavy chain class switching.. J Immunol.

[59] Mosialos G, Birkenbach M, Yalamanchili R, VanArsdale T, Ware C (1995). The Epstein-Barr virus transforming protein LMP1 engages signaling proteins for the tumor necrosis factor receptor family.. Cell.

[60] Gires O, ZimberStrobl U, Gonnella R, Ueffing M, Marschall G (1997). Latent membrane protein 1 of Epstein-Barr virus mimics a constitutively active receptor molecule.. EMBO J.

[61] Li MJ, Maizels N (1999). Activation and targeting of immunoglobulin switch recombination by activities induced by EBV infection.. J Immunol.

[62] Jabara HH, Schneider LC, Shapira SK, Alfieri C, Moody CT (1990). Induction of germ-line and mature C epsilon transcripts in human B cells stimulated with rIL-4 and EBV.. J Immunol.

[63] Cerutti A, Zan H, Schaffer A, Bergsagel L, Harindranath N (1998). CD40 ligand and appropriate cytokines induce switching to IgG, IgA, and IgE and coordinated germinal center and plasmacytoid phenotypic differentiation in a human monoclonal IgM+IgD+ B cell line.. J Immunol.

[64] Gil Y, Levy-Nabot S, Steinitz M, Laskov R (2007). Somatic mutations and activation-induced cytidine deaminase (AID) expression in established rheumatoid factor-producing lymphoblastoid cell line.. Mol Immunol.

[65] Chezar I, Lobel-Lavi L, Steinitz M, Laskov R (2008). Ongoing somatic hypermutation of the rearranged VH but not of the V-lambda gene in EBV-transformed rheumatoid factor-producing lymphoblastoid cell line.. Mol Immunol.

[66] Laskov R, Yahud V, Hamo R, Steinitz M (2011). Preferential targeting of somatic hypermutation to hotspot motifs and hypermutable sites and generation of mutational clusters in the IgVH alleles of a rheumatoid factor producing lymphoblastoid cell line.. Mol Immunol.

[67] Epeldegui M, Hung YP, McQuay A, Ambinder RF, Martinez-Maza O (2007). Infection of human B cells with Epstein-Barr virus results in the expression of somatic hypermutation-inducing molecules and in the accrual of oncogene mutations.. Mol Immunol.

[68] Sugden B, Mark W (1977). Clonal transformation of adult human leukocytes by Epstein-Barr virus.. J Virol.

[69] Nikitin PA, Yan CM, Forte E, Bocedi A, Tourigny JP (2010). An ATM/Chk2-mediated DNA damage-responsive signaling pathway suppresses Epstein-Barr virus transformation of primary human B cells.. Cell Host Microbe.

[70] Cerri M, Capello D, Muti G, Rambaldi A, Paulli M (2004). Aberrant somatic hypermutation in post-transplant lymphoproliferative disorders.. Br J Haematol.

[71] Gaidano G, Pasqualucci L, Capello D, Berra E, Deambrogi C (2003). Aberrant somatic hypermutation in multiple subtypes of AIDS-associated non-Hodgkin lymphoma.. Blood.

[72] Pasqualucci L, Neumeister P, Goossens T, Nanjangud G, Chaganti RS (2001). Hypermutation of multiple proto-oncogenes in B-cell diffuse large-cell lymphomas.. Nature.

[73] Nemerow GR, Wolfert R, McNaughton ME, Cooper NR (1985). Identification and characterization of the Epstein-Barr virus receptor on human B lymphocytes and its relationship to the C3d complement receptor (CR2).. J Virol.

[74] Fingeroth JD, Weis JJ, Tedder TF, Strominger JL, Biro PA (1984). Epstein-Barr virus receptor of human B lymphocytes is the C3d receptor CR2.. Proc Natl Acad Sci U S A.

[75] Shannon-Lowe C, Baldwin G, Feederle R, Bell A, Rickinson A (2005). Epstein-Barr virus-induced B-cell transformation: quantitating events from virus binding to cell outgrowth.. J Gen Virol.

[76] Banchereau J, de Paoli P, Valle A, Garcia E, Rousset F (1991). Long-term human B cell lines dependent on interleukin-4 and antibody to CD40.. Science.

[77] Aubin J, Davi F, Nguyen-Salomon F, Leboeuf D, Debert C (1995). Description of a novel FR1 IgH PCR strategy and its comparison with three other strategies for the detection of clonality in B cell malignancies.. Leukemia.

[78] Montesinos-Rongen M, Schmitz R, Courts C, Stenzel W, Bechtel D (2005). Absence of immunoglobulin class switch in primary lymphomas of the central nervous system.. Am J Pathol.

[79] Brochet X, Lefranc MP, Giudicelli V (2008). IMGT/V-QUEST: the highly customized and integrated system for IG and TR standardized V-J and V-D-J sequence analysis.. Nucleic Acids Res.

[80] Kabat EA, Wu TT, Bilofsky H (1978). Variable region genes for the immunoglobulin framework are assembled from small segments of DNA–a hypothesis.. Proc Natl Acad Sci U S A.

[81] Zhang W, Bardwell PD, Woo CJ, Poltoratsky V, Scharff MD (2001). Clonal instability of V region hypermutation in the Ramos Burkitt's lymphoma cell line.. Int Immunol.

[82] Kelly G, Bell A, Rickinson A (2002). Epstein-Barr virus-associated Burkitt lymphomagenesis selects for downregulation of the nuclear antigen EBNA2.. Nat Med.

