# Supplementary figures and images for: Epstein-Barr Virus Infection of Naïve B Cells In Vitro Frequently Selects Clones with Mutated Immunoglobulin Genotypes: Implications for Virus Biology
Source: PLoS Pathog. 2012 May 10;8(5):e1002697. doi: 10.1371/journal.ppat.1002697 (PMC3349760; doi:10.1371/journal.ppat.1002697)

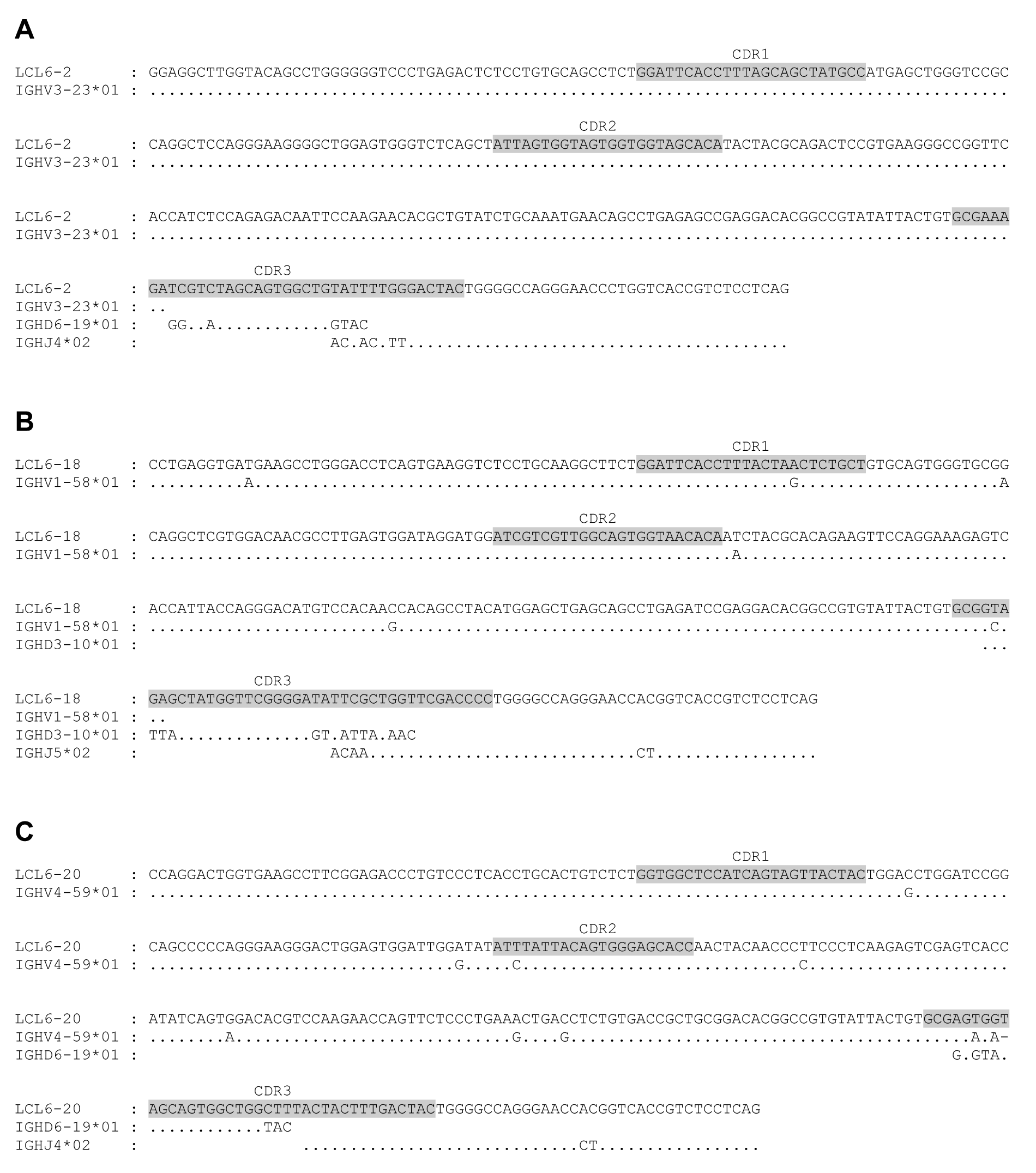

Supplement: Figure S1 — IgH sequences from 3 limiting dilution LCLs established from LCL6. In each case the amplified IgH sequence is aligned with the nearest germline IGHV, IGHD and IGHJ alleles, with sequence identities shown as dots. (A) shows an example of a germline IgH sequence while (B) and (C) show examples of mutated IgH sequences with 5 and 7 changes, respectively, taken from Table 2. Complementarity determining regions CDR1, CDR2 and CDR3 are shaded. Note that the IgVH sequence starts at codon 9 and nucleotide changes in CDR3 are ignored. (TIF) [file ppat.1002697.s001.tif]

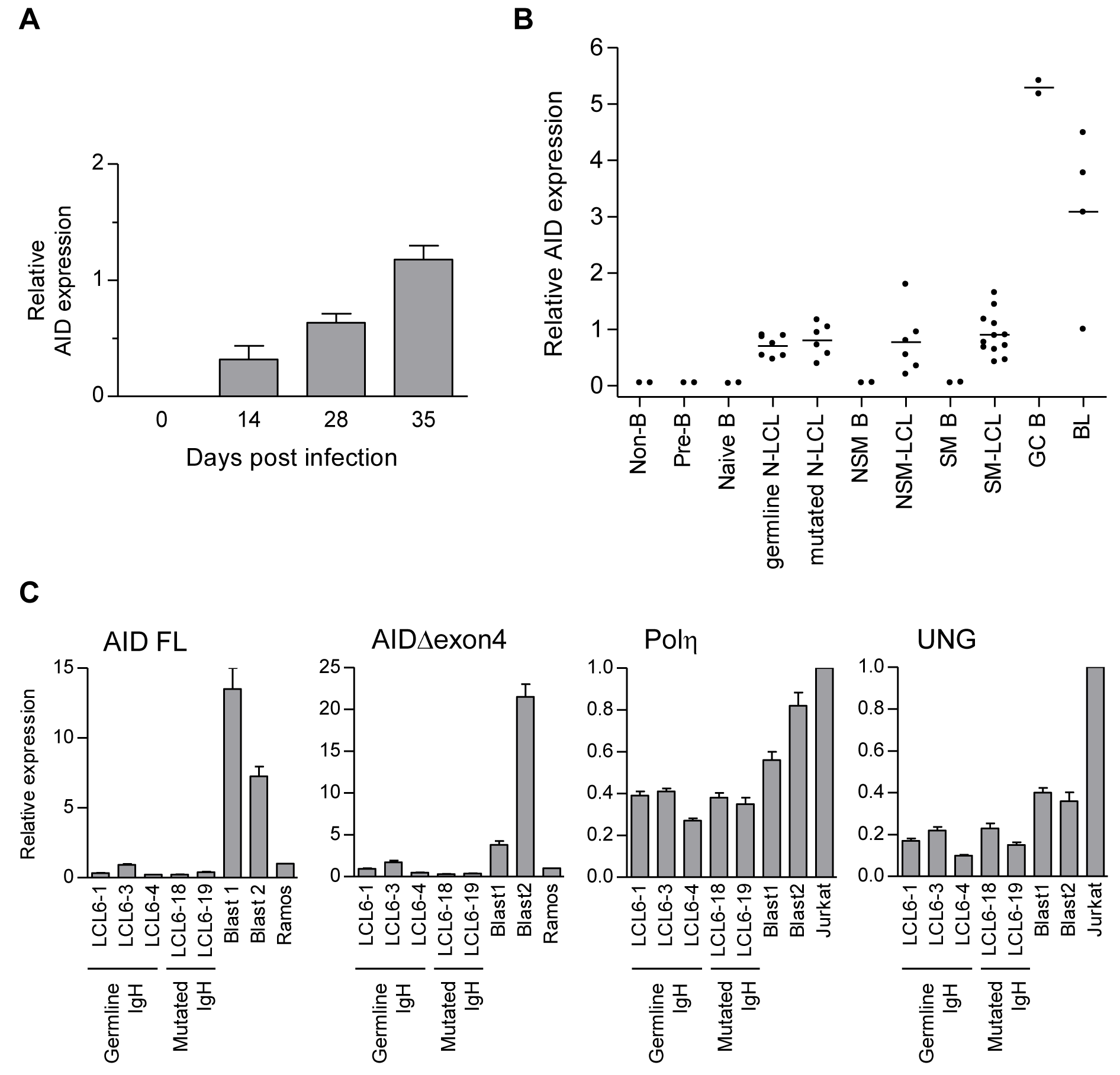

Supplement: Figure S2 — Expression of AID and accessory factors involved in SHM. (A) AID expression was quantified by real time RT-PCR in a bulk LCL culture at various time points post infection. Normalised AID values are expressed relative to the reference BL line Ramos. (B) AID expression quantified by real time RT-PCR in uninfected naïve (N), non-switched memory (NSM) and switched memory (SM) B cells and N-, NSM- and SM-derived limiting dilution LCLs. Data are shown separately for N-derived LCLs with germline or mutated IgH sequences. Two T cell lines (Jurkat and Molt4) and two pre-B cell lines (Nalm6 and Nalm16) were included as negative controls, while four BL lines (Akata-BL, P3HR1-BL, Rael-BL and Ramos-BL) and two germinal centre (GC) B cell preparations served as positive controls. Note that the reference Ramos-BL cell line has the lowest AID expression of the four BL lines. AID values are expressed as in panel A. (C) Expression of full length (FL) AID transcripts, exon4-deleted AID variant transcripts, UNG transcripts and DNA polη transcripts measured by real time RT-PCR. Data are shown from 5 representative naive B cell-derived limiting dilution LCL cultures with either germline or mutated IgH genotypes and 2 polyclonal CD40L/IL4 stimulated N cell-derived B blasts (Blast 1 and Blast 2) from separate donors. Normalised values are expressed relative to Ramos-BL (AID) or Jurkat cells (UNG and DNA polη). Data are the mean (+/− SD) of triplicate readings. (TIF) [file ppat.1002697.s002.tif]

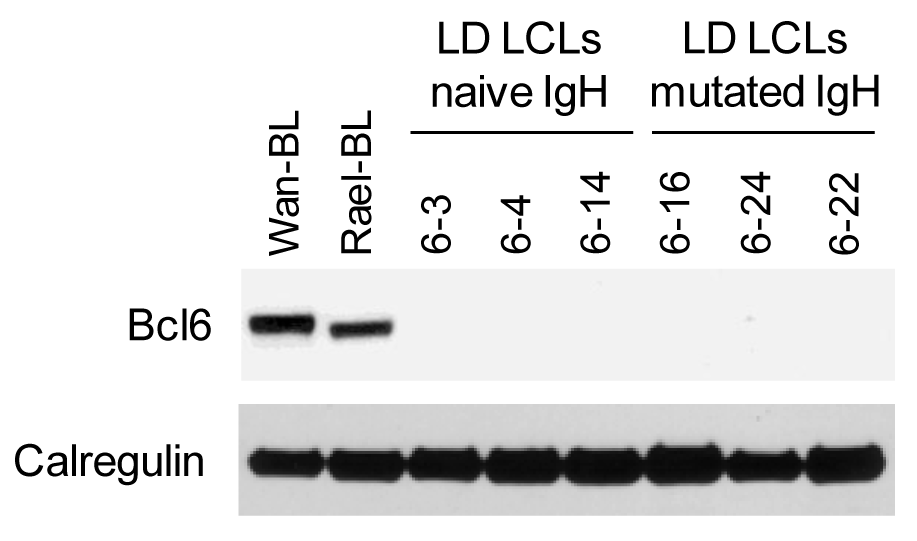

Supplement: Figure S3 — Bcl6 expression is downregulated following EBV transformation. Immunoblots show expression of Bcl6 in representative LD-LCL cultures carrying either germline or mutated IgH genotypes. Two EBV-positive BL cell lines (Wan-BL, Rael-BL) were included as positive controls for Bcl6 expression. Calregulin was used as a loading control. (TIF) [file ppat.1002697.s003.tif]

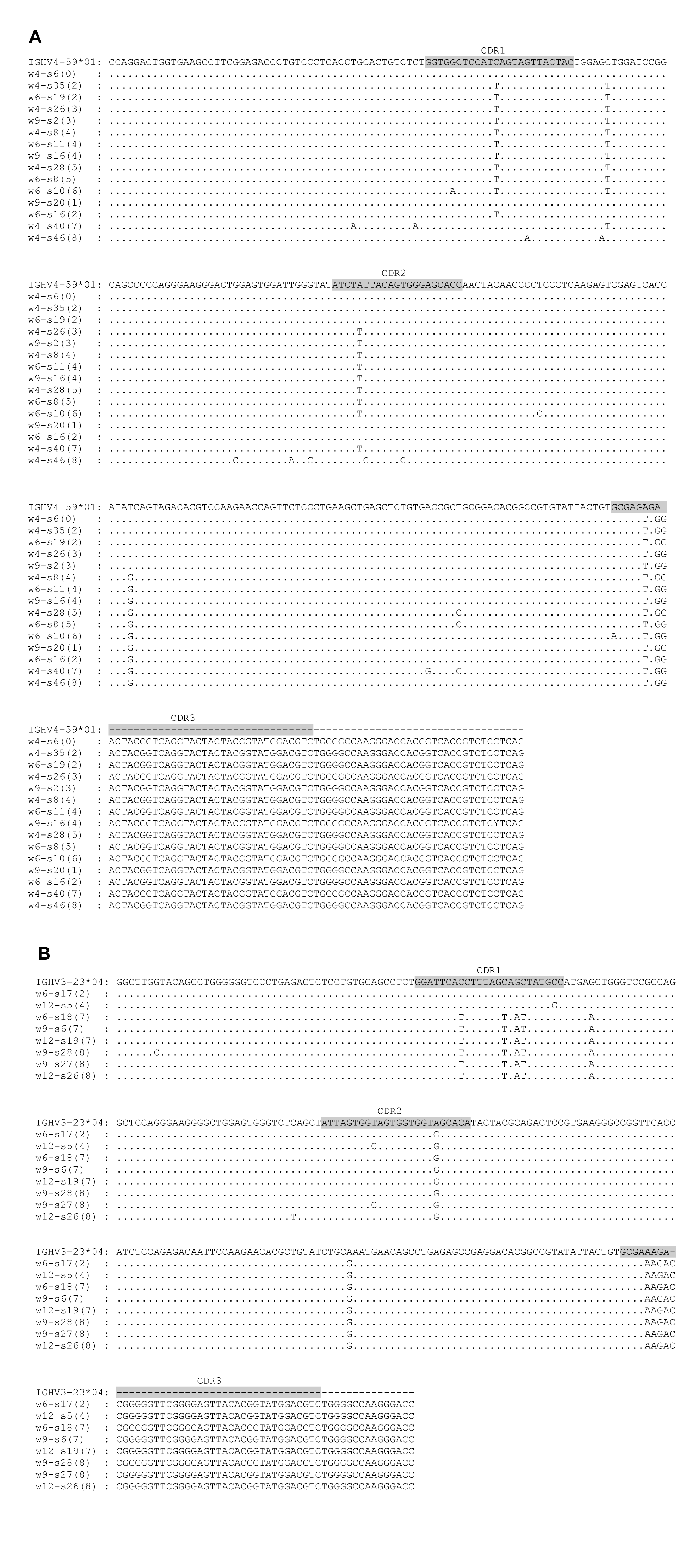

Supplement: Figure S4 — Examples of clonal variation within LCL bulk cultures. Shown are selected clonally-related IgH sequences amplified from naive B cell derived bulk LCL cultures which were used to construct the genealogical trees shown in Figure 8. The sequence name reflects the time post infection (in weeks, w) at which the clone was isolated and the sequence number (s), while the number in parentheses indicates the number of mutations relative to the nearest germline sequence shown on the top line. Sequence identities are shown as dots; complementarity determining regions CDR1, CDR2 and CDR3 are shaded. (A) shows the results for LCL5 while (B) shows the results for LCL8. (TIF) [file ppat.1002697.s004.tif]
