# Supplementary material for: Epstein-Barr Virus Infection of Naïve B Cells In Vitro Frequently Selects Clones with Mutated Immunoglobulin Genotypes: Implications for Virus Biology
Source: PLoS Pathog. 2012 May 10;8(5):e1002697. doi: 10.1371/journal.ppat.1002697 (PMC3349760; doi:10.1371/journal.ppat.1002697)
Supplement: Table S1 — Bcl6 MMC mutation status in naive B cell-derived LD LCL clones with mutated IgH genotypes. (DOC) [file ppat.1002697.s005.doc]

**Supplementary Table S1.** Bcl6 MMC mutation status in naive B cell-derived LD LCL clones with mutated IgH genotypes

| **Cell type** | **Clonotypes tested** | **Mutated Bcl6 sequences (%)** | **Number of mutations** |
| --- | --- | --- | --- |
| IgD^+^ CD27^-^ naive | 18 | 1 (5.5) | 1 x 1 |
| CD27^+^ memory | 24 | 10 (41.6) | 7 x 1, 2 x 2, 1 x 3 |
| N-derived LD LCLs with mutated IgH | 18 | 2 (11.1) | 1 x 2, 1 x 3 |
